# Crystal structure, stability, and transport properties of Li_2_BeAl and Li_2_BeGa Heusler alloys: a DFT study

**DOI:** 10.1038/s41598-024-63092-x

**Published:** 2024-05-28

**Authors:** Sima Mahmoudi, Mir Maqsood Golzan, Ebrahim Nemati-Kande

**Affiliations:** 1 Department of Physics, Faculty of Sciences, Urmia University, Urmia, Iran; 2 Department of Physical Chemistry, Chemistry Faculty, Urmia University, Urmia, Iran

**Keywords:** Heusler, Li_2_BeAl, Li2BeGa, DFT, Boltzmann transport theory, Physical chemistry, Chemical physics

## Abstract

In this study, the structural, elastic, electronic, and thermoelectric properties of full Li_2_BeAl and Li_2_BeGa Heusler alloys were explored using density functional and the Boltzmann transport theories. The GGA and HSE approximations have been used for the exchange–correlation potential. Results indicated that these two compounds are more energetically stable in the inverse Heusler structure. Additionally, both Li_2_BeAl and Li_2_BeGa Heusler alloys were found to be mechanically stable due to the positive values of the elastic constants. Also, the high values of the Young's modulus indicate that these compounds are stiff and exhibit a semi-metallic nature. The band gaps were determined to be 0.13 eV and − 0.22 eV for Li_2_BeAl and Li_2_BeGa alloys, respectively, using the GGA approximation. By employing the HSE hybrid functional, however, the band gap for Li_2_BeAl increased to 0.26 eV, and for Li_2_BeGa, it decreased to − 0.16 eV. Regarding thermoelectric properties, Seebeck coefficient, electrical conductivity, electronic and lattice thermal conductivities, power factor, and the figure of merit have been calculated for both Li_2_BeAl and Li_2_BeGa Heusler alloys at different temperatures. Seebeck coefficient in both alloys decreases with increasing the temperature and has the highest value at 300 K. Thermal conductivity and electrical conductivity increase with increasing the temperature, which confirms the intermetallic behavior of the Heusler alloys. The results obtained for both alloys show that *n*-type doping has better thermoelectric properties than *p*-type doping. The maximum value of the figure of merit (ZT) was obtained for n-type doping, which was 1.43 at 660 K for Li_2_BeAl and 0.39 at 1000 K for Li_2_BeGa alloy. The high values of ZT especially for electron-dopped Li2BeAl suggest the great potential of this material for use in thermoelectric devices. This study suggests that the proposed materials have potential applications in spintronic devices and thermoelectric materials due to their intermetallic character and effective thermoelectric coefficients.

## Introduction

Heusler discovered the first alloy of the Heusler type, Cu_2_MnAl, in 1903^1^. However, since Heusler compounds are made up of non-ferromagnetic materials, their interesting ferromagnetism has kept them a scientific wonder for a long time. Heusler compounds became intriguing materials for a variety of outstanding functional applications in the twenty-first century, including thin-film growth, spintronic (giant magnetoresistance) technologies, thermoelectric and other energy conversion systems, and novel characteristics^2^. Regular ternary Heusler compounds come in two types: full Heusler with X_2_YZ and half Heusler with XYZ chemical formulas^3^. Normally, X and Y can be transition elements, and Z is one of the main group elements. Heusler alloys are also expanded to binary and quaternary complexes as a result of chemical replacement and structural change^2^.

The depletion of the fossil resources, economic growth, and environmental concerns have compelled scientists to look for fresh renewable energy sources. The introduction of new thermoelectric materials for energy transformation in small-scale energy production and refrigeration devices, has been the key driving force behind this search. Due to the potential applications of the ternary Heusler complexes, the Li atom in the form of half-Heusler compounds have recently gained a lot of attention in the creation of thermoelectric devices, spintronics, optoelectronics, and other technologies^4^. Kalarasse et al.^4^ theoretically studied the elastic and electronic properties of alkali pnictide compounds Li_3_Sb, Li_3_Bi, Li_2_NaSb, and Li_2_NaBi. Dogan et al.^1^ studied the lattice dynamics and electronic properties of Li_2_AlGa and Li_2_AlIn Heusler alloys. Mesbah et al.^5^ theoretically studied the structural, electronic, elastic, and optical properties of full Heusler compounds of Li_2_CaC and Li_2_SrC. The electrical and thermoelectric properties of half-Heusler alloys LiSrN, LiSrP, and LiSrAs were reported by Benazouzi et al.^6^. Joshi et al.^7^ theoretically investigated the optical and elastic properties of semi-Heusler MCoSb (M = Ti, Zr, and Hf) compounds. The obtained results showed that the bulk modulus decreases with increasing temperature and increases with increasing pressure for all three compounds. The study of the optical properties of the compounds showed high reflectance in the infrared region of the photon energy. The imaginary part of the dielectric function confirms the non-metallic nature of these compounds.

The structural, electronic, and magnetic properties of Co_2_MnGe and Co_2_MnSn ternary Heusler compounds were investigated by Rai et al.^8^. The calculated density of states and band structures showed that Co_2_MnGe and Co_2_MnSn exhibit semi-metallic ferromagnetism characteristics.

Khelfaoui et al.^9^ considered the structural, elastic, electronic, and magnetic properties of Heusler compounds of Zr_2_PdZ (Z = Al, Ga, and In) using DFT methods in the GGA approximation. The band structure analysis for these compounds showed that all three compounds have almost semi-metallic properties with a narrow indirect band gap in the minority spin channel of 0.36 eV, 0.46 eV, and 0.40 eV for Zr_2_PdAl, Zr_2_PdGa, and Zr_2_PdIn, respectively. Mohanta et al.^5^ also calculated the electronic structure and magnetic properties of Cr-based inverse Heusler alloys using the linear enhanced plane wave DFT method. They concluded that, these materials were promising options for semi-metallic magnetic systems that can be used for spintronics and STM scanner tip fabrication. Maafa et al.^10^ also explored the electronic properties of some new Heusler alloys with various magnetic applications. Among the compounds, only two Co_2_ZrGe and Fe_2_ZrSb structures exhibited an indirect band gap of 0.59 eV and 0.73 eV in the minority spin channel, and were recognized as real semi-metals.

Forozani et al.^11^ theoretically reported the structural, electronic, magnetic, and vibrational properties of all Heusler Ir_2_CrSi and Ir_2_CrGe compounds. The total magnetic moment for Ir_2_CrSi and Ir_2_CrGe were calculated to be 3.94 μB and 3.92 μB, respectively. The composition of Ir_2_CrSi exhibits semi-metallic properties with a band gap of 0.105 eV. The lattice constant of semi-metallic Ir_2_CrSi exhibits 100% spin polarization at the Fermi level, while Ir_2_CrGe has 99% spin polarization at the Fermi level, making these alloys suitable for spintronic devices. Lahoupour et al.^12^ investigated the Co_2_FeAl/AlN using DFT methods. Studies of the composition of Co_2_FeAl showed that it is a semi-metal that has 100% polarization at the Fermi level. Kumar et al.^13^ reported the magnetic, optical, and electronic properties of Y_2_FeSi perfect Heusler alloys using ab initio calculations. Y_2_FeSi compound exhibited half ferromagnetic behavior with 75% spin polarization at the Fermi level, and the obtained optical properties showed that this compound is suitable for optical and spintronic applications.

The structural, thermoelectric, half-metallic, optical, and thermoelectric properties of different classes of perovskites such as A_2_BCl_6_(A = Cs, K and B = Se, Sn, Ta, Te, Ti, W, Zr, Mn, Mo, Os, Pd, Pt, Re, Ru, Tc, W),^14^ Cs_2_ABF_6_ (AB = BiAu, AgIr, CuBi, GaAu, InAs, InAg, InAu, InSb and InBi),^15^ Sr_2_EuReO_6,_^16^ Ba_3_B(Nb,Ta)_2_O_9_ (B = Mg, Ca, Sr, Cd, Hg, Zn, Fe, Mn, Ni, and Co),^17^ XZrO_3_ (X = Ca, Sr, Ba),^18^ SrMO_3_ (M = Hf and Pt),^19^ AgBeX_3_ (X = F and Cl),^20^ AGeF_3_ (A = Ga and In),^21^ LiRCl_3_ (R = Be and Mg),^22^ NaMF_3_ (M = Si and Ge),^23^ AlRF_3_ (R = N, P),^24^ and BWF_3_ (W = S and Si)^25^ were recently investigated by different researchers using DFT methods. These compounds were suggested to be good candidates for using in thermoelectric, spintronic, optical, energy storage, and optoelectronic applications. Khan et al.^26^ also conducted DFT studies on ZnO nanoparticles, which show that this compound doped with Ce and Co has interesting magnetic and dielectric properties, which makes it suitable for use in spin-based electronic devices as well as the memory devices for memory applications, and it seems that the resistive random access is the best choice.

Recently, Heusler quaternary alloys have also received the attention of researchers. Idrissi et al.^27^ investigated the magnetic and electronic properties of Heusler quaternary alloys of Co. The results of the band structure and density of states (DOS) for Heusler alloys CoYCrZ (Z = Si and Ge) exhibited that these compounds display semi-metallic behavior. Andharia and coleague^28^ reported different properties of CrTiCoZ (Z = Al/Si) quaternary Heusler alloys. The electronic structure calculations revealed that these compounds are semi-metals. CrTiCoAl exhibits a direct band gap of 0.15 eV and a magnetic moment of 2 μB. However, an incorrect magnetic moment of 0.99 μB was calculated for CrTiCoSi. The researchers also suggested that these two Heusler compounds can be used as spin injectors in spintronic devices. Berri^29^ also investigated structural, thermoelectric, and the elastic properties of half-metallic CoCrScZ (Z = Al, Si, Ge, and Ga) quaternary Heusler alloys. Berri^29^ found that CoCrScAl, CoCrScGe, and CoCrScGa compounds can be used in spin injection applications.

In this work, quantum DFT and Boltzmann transport theory methods were used to explore the Li_2_BeAl and Li_2_BeGa full Heusler alloys. The crystal structure of these two full Heusler alloys was optimized, and the phonon dispersion density of states of the optimized structures were calculated to ensure the mechanical stability of the relaxed crystal unit cells. The ab-initio molecular dynamics calculations were also used to explore the thermal stability of the compounds even at the high temperature of 1000 K. Different physical properties of these two full Heusler alloys such as structural, dynamical, electronic, optical, thermal, and thermoelectric properties were then calculated to explore their potential applications.

## Methodology

In this study, the electronic and thermoelectric properties of Li_2_BeAl and Li_2_BeGa full Heusler alloys were calculated. The Quantum Espresso (QE) package^30^ was implemented in all electronic calculations by implementing the generalized gradient approximation (GGA) based on the correlation exchange function of Perdew, Burke, and Ernzerhof (PBE)^31^ and the hybrid functional of Heyd-Scuseria-Ernzerh (HSE)^32^. Also, Thermo-pw^33^ package was used to calculate network dynamics and to calculate the phonon scattering curves and phonon density of states. In addition, The semi-classical Boltzmann transport equations and generating maximally-localized Wannier functions (MLWF),^34^ as implemented in Boltztrap2^35^ and Wannier90^36^ computational packages, were used to investigate thermoelectric properties such as the Seebeck coefficient, thermal conductivity, and electrical conductivity. Xcrysden^37^ software was also used to visualize crystal structures.

The kinetic energy cut-off of the wave function was set to be 80 Ry and a 350 Ry cutoff energy was used for the kinetic energy. Also, the lattice structures were optimized with the energy uncertainty of 1 × 10^–10^ Ry. The first Brillouin zone was modeled using a 24 × 24 × 24 k-point mesh. For calculation of the phonon dispersion curves and the density of phonon states, we employed a q-point mesh of 16 × 16 × 16 using Thermo-pw code. Additionally, we used an 8 × 8 × 8 K-point mesh in the Wannier 90 code to calculate the thermoelectric properties. The Boltzmann density of states were obtained with the k-mesh of 300, and the energy steps of 1 × 10^–4^ was used. A dense k-mesh of 24 × 24 × 24 has also been used to calculate the thermoelectric properties using Boltztrap2 code.

Dynamical stability of the studied alloys was studied using Car-Parrinello^38^ ab-initio molecular dynamics (CPMD) simulation in canonical ensemble (NVT) formalism, as implemented in QE. Calculations are performed on a 2 × 2 × 2 supercell containing 32 atoms at temperatures of 500 and 1000 K. Nose–Hoover thermostat was used to control the temperature, and the time-step of 4 a.u. with a total run of 55,000 steps was done.

The lattice thermal conductivity of the crystals was also calculated using the finite displacement method in a relaxation time approximation solution of the linearized Boltzmann transport equation as implemented in Phono3py^39^ code in the DFT framework on 2 × 2 × 2 supercells. Total of 452 supercells were produced by Phono3py without any cut-off, and the second and third order interatomic force constants are calculated using QE with 5 × 5 × 5 k-point mesh. A 50 × 50 × 50 q-mesh then was used to calculate the lattice thermal conductivity using Phono3py code.

## Results and discussion

### Structural properties

**Figure 1:**
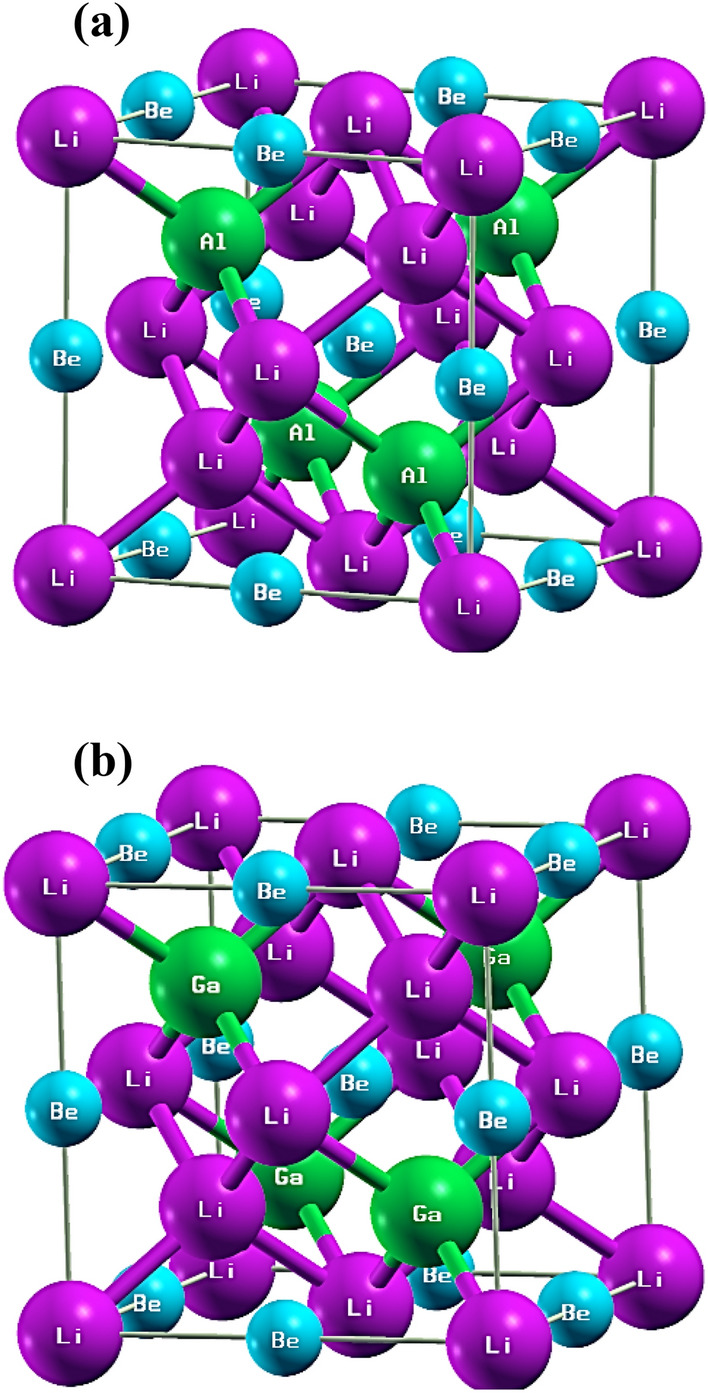
The relaxed inverse Heusler structures of (**a**) Li_2_BeAl and (**b**) Li_2_BeGa Heusler compounds.

**Figure 2:**
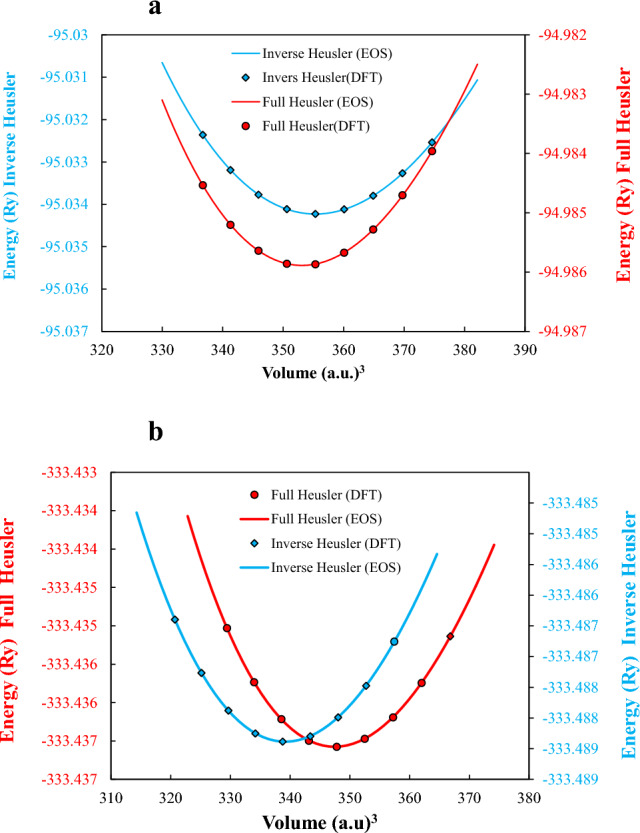
Energy-Volume optimization of the lattice parameters of (**a)** Li_2_BeAl and (**b)** Li_2_BeGa alloys.

Full Heusler alloys, which have the X_2_YZ chemical formula, exhibit an L2_1_ crystal structure with a space group of ${\text{Fm}}\overline{{3}} {\text{m}}$ (225). The X, Y, and Z elements occupy the Wyckoff positions of (0, 0, 0) and (1/2, 1/2, 1/2), (1/4, 1/4, 1/4), and (3/4, 3/4, 3/4), respectively. If the positions of the X and Y elements are exchanged in the Full Heusler composition, the resulting compound is known as an inverse Heusler composition^2^. Inverse Heusler compounds have the space group of ${\text{F}}\overline{4} {\text{3m}}$ and XA structure (216). The crystal structures of the relaxed Li_2_BeAl and Li_2_BeGa Heusler compounds were shown in Fig. 1. To determine the stable lattice structure and equilibrium constants of Li_2_BeAl and Li_2_BeGa, the calculated total energies as a function of lattice volume are obtained for both full Heusler structures and inverse Heusler structures. Then, the total energy (E) as a function of the primitive unit cell volume (V) was fitted with the third-order Birch-Murnaghan equation of state (EOS)^40^:

1 $$E\left( V \right) = E_{0} + {{B\left( {V\left( {{{V_{0} } \mathord{\left/ {\vphantom {{V_{0} } V}} \right. \kern-0pt} V}} \right)^{{B^{\prime}}} - V_{0} } \right)} \mathord{\left/ {\vphantom {{B\left( {V\left( {{{V_{0} } \mathord{\left/ {\vphantom {{V_{0} } V}} \right. \kern-0pt} V}} \right)^{{B^{\prime}}} - V_{0} } \right)} {\left( {B^{\prime}\left( {B^{\prime} - 1} \right)} \right)}}} \right. \kern-0pt} {\left( {B^{\prime}\left( {B^{\prime} - 1} \right)} \right)}} + {{B\left( {V - V_{0} } \right)} \mathord{\left/ {\vphantom {{B\left( {V - V_{0} } \right)} {B^{\prime}}}} \right. \kern-0pt} {B^{\prime}}}$$

to estimate the total energy (*E*_0_) and the volume of the equilibrium primitive cell (V_0_), as well as the bulk modulus (B) and its derivative (B′). The fitted and DFT results were shown in Fig. 2, and the calculated parameters are reported in Table 2. From Fig. 2, it is evident that the Birch-Murnaghan EOS was well fitted to the DFT data, and therefore, the obtained parameters are consistence. Furthermore, it is seen that the inverse Heusler structure exhibits a lower total energy, which indicates that it is a more stable and favorable structure for both Li_2_BeAl and Li_2_BeGa Heusler alloys.

Cohesive energy^41^ (*E*_coh_) is the total energy of a single atoms arranged in a solid state. It enables the analysis of the structural stability of a compound following experimental synthesis. The following equation is used to calculate the cohesive energy, for a compound with the X_2_YZ chemical formula:

2 $$E_{coh}^{{X_{2} YZ}} = {{\left( {E_{total}^{{X_{2} YZ}} - \left( {2E_{atom}^{X} + E_{atom}^{Y} + E_{atom}^{Z} } \right)} \right)} \mathord{\left/ {\vphantom {{\left( {E_{total}^{{X_{2} YZ}} - \left( {2E_{atom}^{X} + E_{atom}^{Y} + E_{atom}^{Z} } \right)} \right)} 4}} \right. \kern-0pt} 4}$$

where, *E*_*total*_ is the total energy of the structure, and $E_{atom}^{X}$, $E_{atom}^{Y}$, and $E_{atom}^{Z}$ are the total energy per atom for X (Li), Y (Be), and Z (Al or Ga), respectively. Table 1 represents the total energy values and energy per atom for Li_2_BeAl and Li_2_BeGa Heusler alloys. The negative cohesive energy values confirm that these phases are thermodynamically stable from an energetic standpoint. Also, Li_2_BeAl is slightly more stable than the Li_2_BeGa due to the more negative *E*_*coh*_ value.

**Table 1:** The total energy (E_total_), cohesive energy (E_coh_) and the energies of each individual atom. All energies are in eV.

structure	$E_{total}^{{X_{2} YZ}}$	$E_{atom}^{X}$	$E_{atom}^{Y}$	$E_{atom}^{Z}$	$E_{coh}^{{X_{2} YZ}}$
Li_2_BeAl	− 1293.00	− 195.00	− 357.87	− 533.72	− 2.85
Li_2_BeGa	− 4537.33	− 195.00	− 357.87	− 3778.49	− 2.74

### Dynamical stability

**Figure 3:**
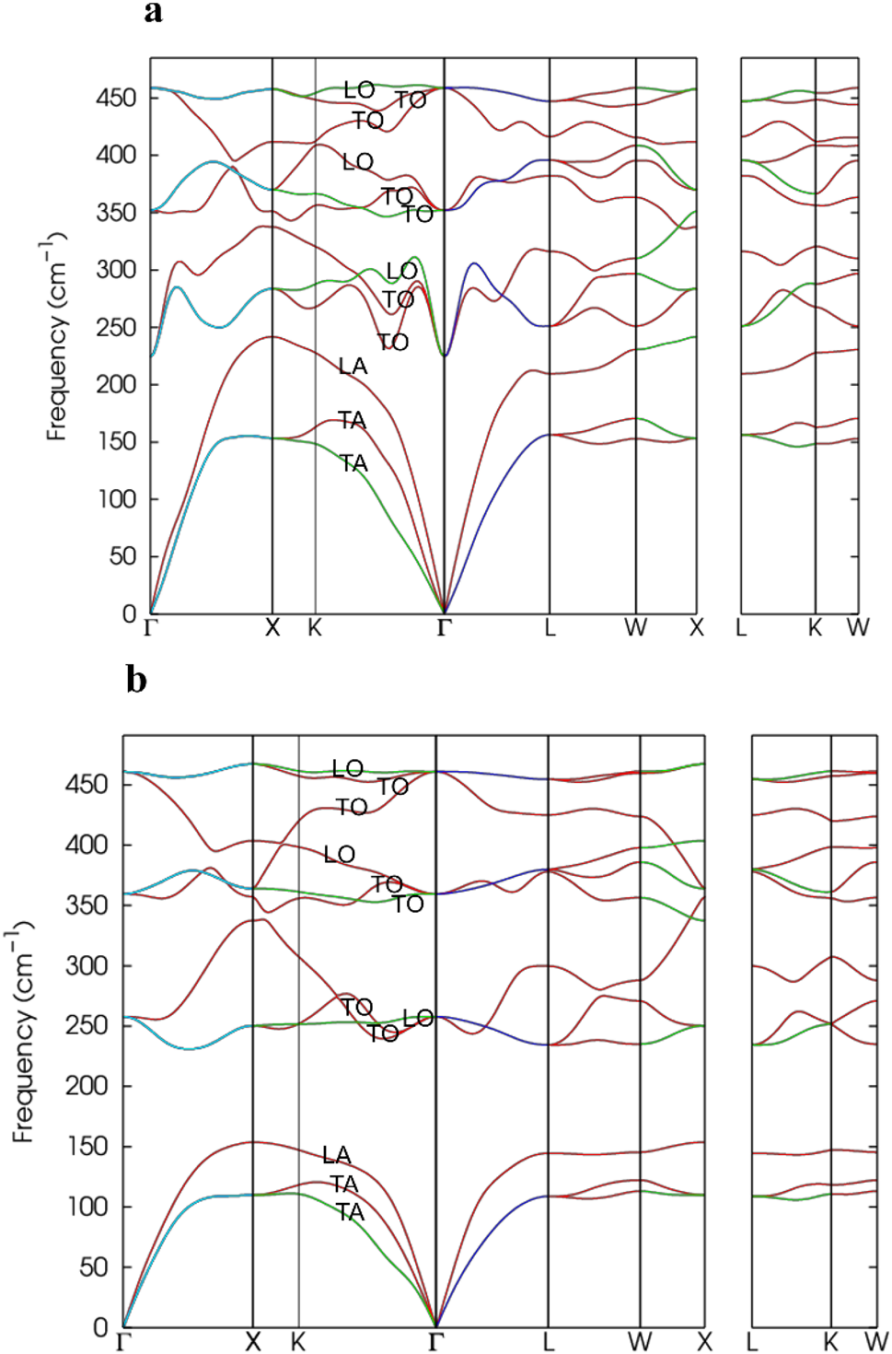
Calculated phonon dispersion curve of (**a)** Li2BeAl, (**b)** Li_2_BeGa Heusler alloys.

**Figure 4:**
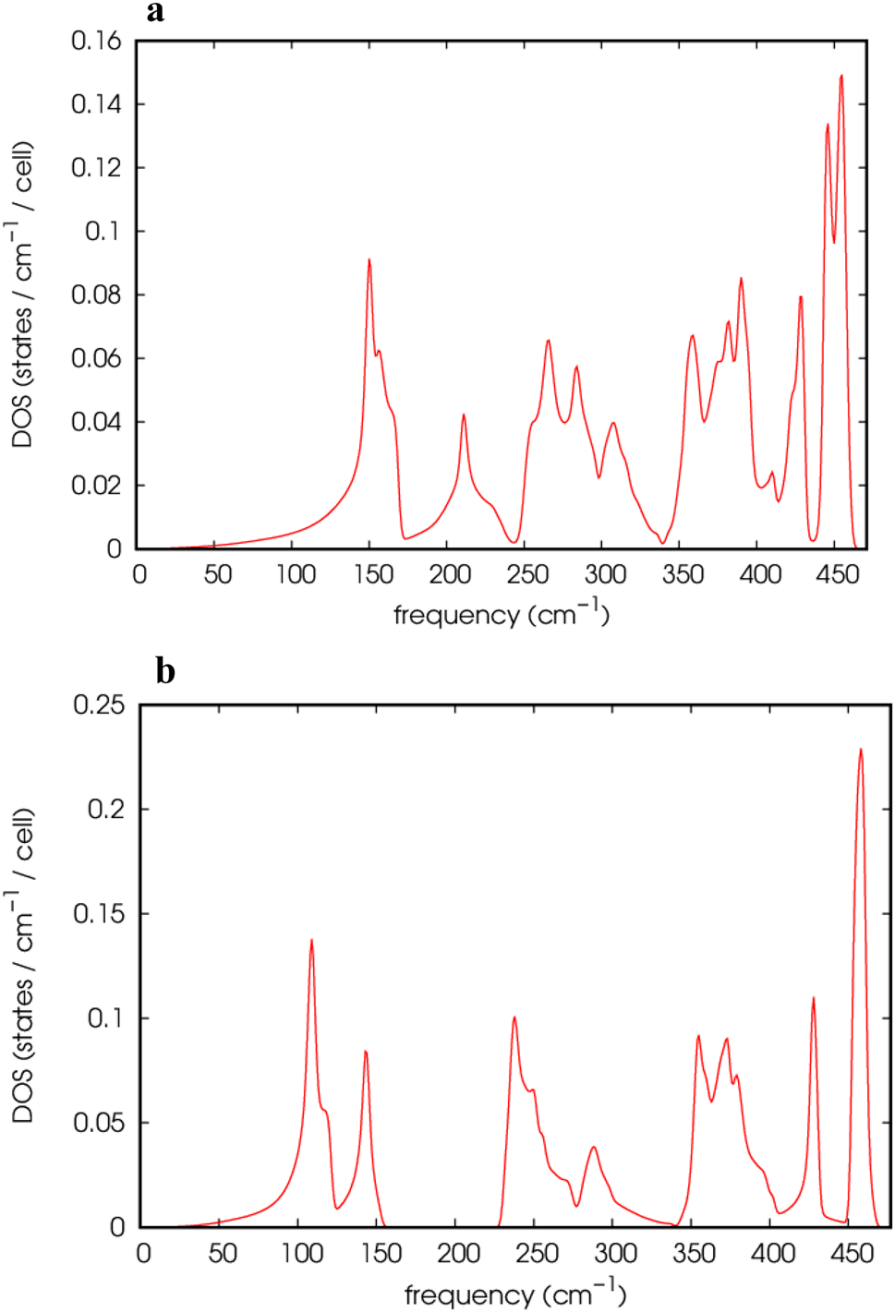
Calculated phonon density of states of (**a)** Li_2_BeAl and (**b)** Li_2_BeGa Heusler alloys.

To investigate the dynamical stability of Li_2_BeAl and Li_2_BeGa Heusler alloys, we also calculated the phonon dispersion curves and phonon density of states. Since Heusler alloys have four atoms per unit cell, each wave vector has twelve phonon states, including three phonon states, three acoustic states, and nine optical states. Figure 3 displays the phonon scattering curves, and Fig. 4 shows the phonon density of states of Li_2_BeAl and Li_2_BeGa Heusler alloys.

Acoustic modes with an energy value of 0 cm^-1^ are considered as phonon modes. Two modes with a lower energy value are transverse acoustic (TA) modes, and one mode with a higher energy value is longitudinal acoustic (LA)^42^. Moreover, optical modes with lower energy values in each branch are transverse optical (TO) modes, while modes with higher energy values in each branch are longitudinal optical (LO) modes^1^. It can be seen from Figs. 3 and 4 that, in both Heusler alloys there are two distinct band regions, and the acoustic bands were completely separated from the optical bands. In other words, the phonon-photon scattering is not permitted in both studied Heusler compounds.

Phonons, especially the TA modes, can be utilized to analyze a compound's thermal conduction properties. If the lowest frequency TO mode has a lower frequency than the TA mode, the thermal conductivity falls. Since low-frequency TO modes scatter significant amounts of TA modes, they reduce the material's ability to conduct heat^43^. In Fig. 3, we observed that there is no interaction between the LA and TO modes; hence, they do not affect the thermal conductivity of Li_2_BeAl and Li_2_BeGa Heusler compounds. Furthermore, the phonon spectra obtained for the Li_2_BeAl and Li_2_BeGa Heusler alloys in all directions exhibit a positive phonon frequency in the Brillouin zone, indicating that these compounds are dynamically stable in the inverse Heusler structure.

### Thermal stability

**Figure 5:**
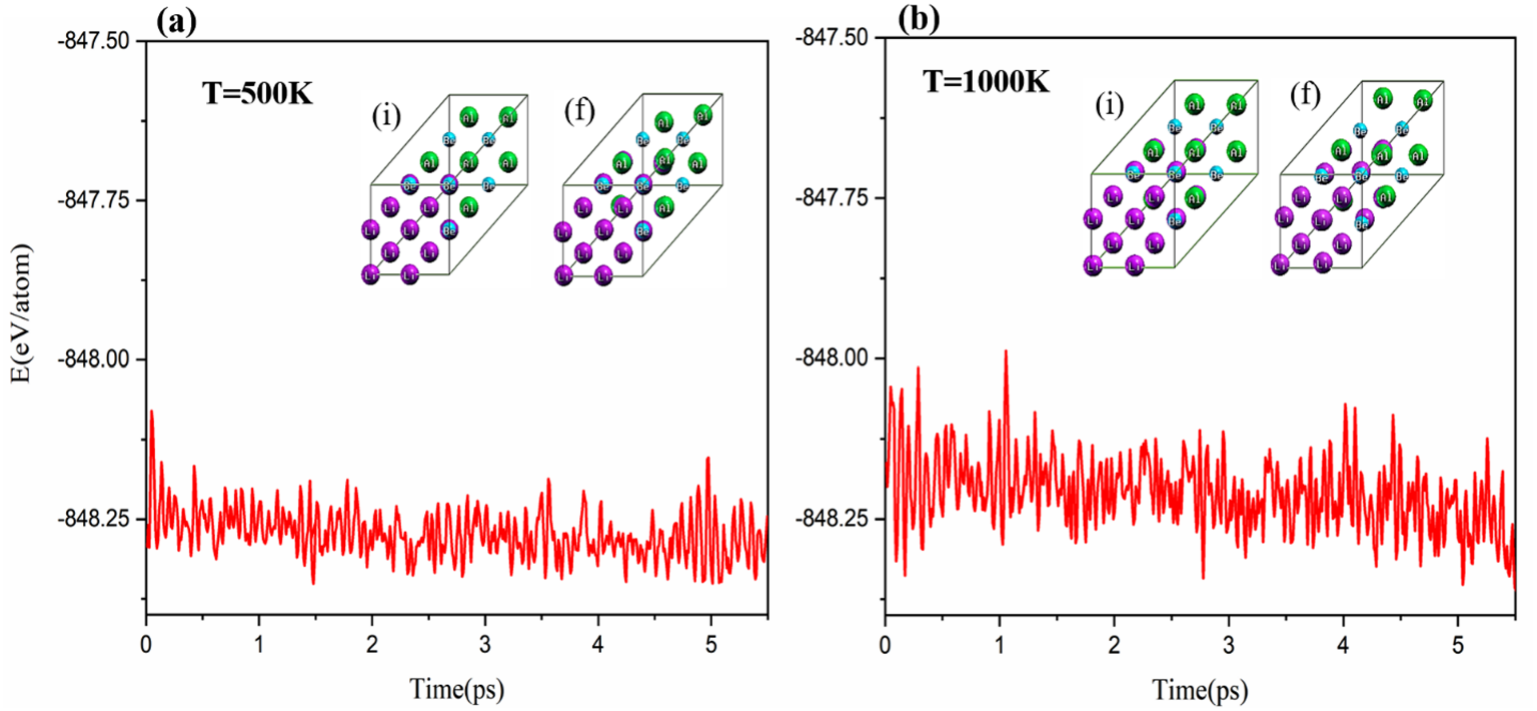
Molecular dynamics (MD) simulation of Li_2_BeAl Heusler alloys at temperatures of 500 K and 1000 K, *i* and *f* show the initial and final geometric structure of Li_2_BeAl Heusler alloys.

**Figure 6:**
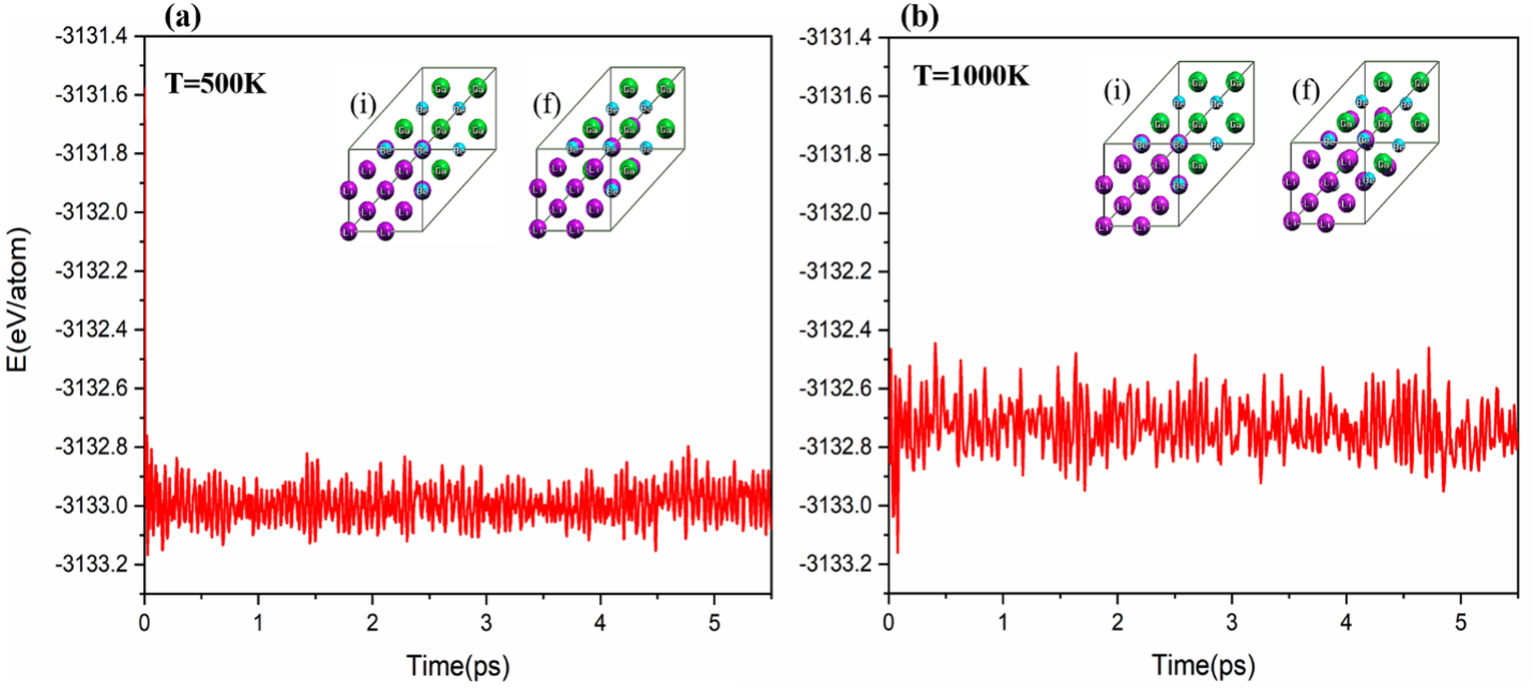
Molecular dynamics (MD) simulation of Li_2_BeGa Heusler alloys at temperatures of 500 K and 1000 K, *i* and *f* show the initial and final geometric structure of Li_2_BeAl Heusler alloys.

In previous sections, thermodynamic stability and dynamic stability were proved for Li2BeAl and Li2BeGa Heusler alloys. In this section, the thermal stability of the 2 × 2 × 2 supercells of Li2BeAl and Li2BeGa Heusler alloys at the temperatures of 500 and 1000 K was performed using CPMD calculations in canonical ensemble (NVT) formalism. It can be seen from Figs. 5 and 6 that, the although the final geometrical structure of the alloys after the simulation at different temperatures shows small distortion compared to the initial structure, the cell structure was remained almost unchanged. Also, in Figs. 5 and 6, it can be seen that the total energy fluctuations have a constant pattern over time. Therefore, Li2BeAl and Li2BeGa Heusler alloys have high thermal and structural stability even at high temperature 1000 K.

### Elastic properties

Elastic properties of the studied compounds were also calculated to check the mechanical stability, ductility, and hardness of Li_2_BeAl and Li_2_BeGa Heusler alloys. There are three independent elastic constants C_11_, C_12_, and C_44_ for a cubic crystal which are calculated using the thermos-pw package^33^, and are listed in Table 2 at T = 0 K and P = 0 Gpa. The Born-Huang^44,45^ mechanical stability criteria for cubic crystals are: C_11_–C_12_ > 0, C_11_ > 0, C_44_ > 0, C_11_ + 2C_12_ > 0 and C_12_ < B < C_11_. All calculated values for these constants are positive for both compounds and are consistent with Born-Huang stability conditions. Therefore, Li_2_BeAl and Li_2_BeGa Heusler alloys are mechanically stable and resistant to deformation and hardness.

**Table 2:** The calculated lattice parameter (a_0_), elastic constants (C_ij_), Bulk modulus (B), Young modulus (E), Shear modulus (G), Poisson’s ratio (ν), density (ρ), Debye temperature, as well as the bulk and shear sound velocities of Li_2_BeAl and Li_2_BeGa Heusler alloys**.**

Property/Compound	Li_2_BeAl	Li_2_BeGa
a_0_(Å)	5.95	5.86
C_11_	96.89	95.09
C_12_	29.12	32.17
C_44_	59.21	66.03
B(GPa)	51.71	53.14
E(Gpa)	108.77	112.42
G(GPa)	47.33	49.03
ν	0.15	0.15
Compressional sound velocity (m/s)	8544.15	6223.83
Bulk sound velocity (m/s)	5733.97	4167.50
Shear sound velocity (m/s)	5485.71	4003.26
Debye temperature (K)	754.05	555.59
Debye sound velocity (m/s)	5979.96	4338.28
*ρ* (g/cm^3)	1.57	3.06

Also, Young's modulus (*E*), bulk modulus (*B*), shear modulus (*G*), and Poisson's ratio (*ν*) were obtained using the Voigt–Reuss–Hill average^46,47^, and were listed in Table 2. The bulk and shear modulus were measured to check the resistance of a crystalline structure to the volume stresses and plastic deformation, respectively. The bulk modulus values were 51.7 and 53.14 GPa for Li_2_BeAl and Li_2_BeGa compounds, respectively. Young's modulus is one of the main elastic constants that measure the stiffness of materials, which were obtained to be 108.77 and 112.41 GPa for Li_2_BeAl and Li_2_BeGa Heusler alloys, respectively. The high values of the Young's modulus indicate the existence of a strong covalent bond as well as the stiffness of the compounds. The Poisson's ratio (*ν*) is a dimensionless material property that predicts how much a material will laterally contract when experiencing longitudinal strain. The Poisson's ratio has a value between 0 and 0.5. If ν of a compound is close to zero, the composition does not deform elastically, and if it is close to 0.5, the composition has a large elastic deformation. The value of Poisson's ratio for Li_2_BeAl alloy is about 0.15 and for Li_2_BeGa alloy is 0.146, which shows that both compounds may not deform elastically and have brittle behavior.

### Electronic properties

**Figure 7:**
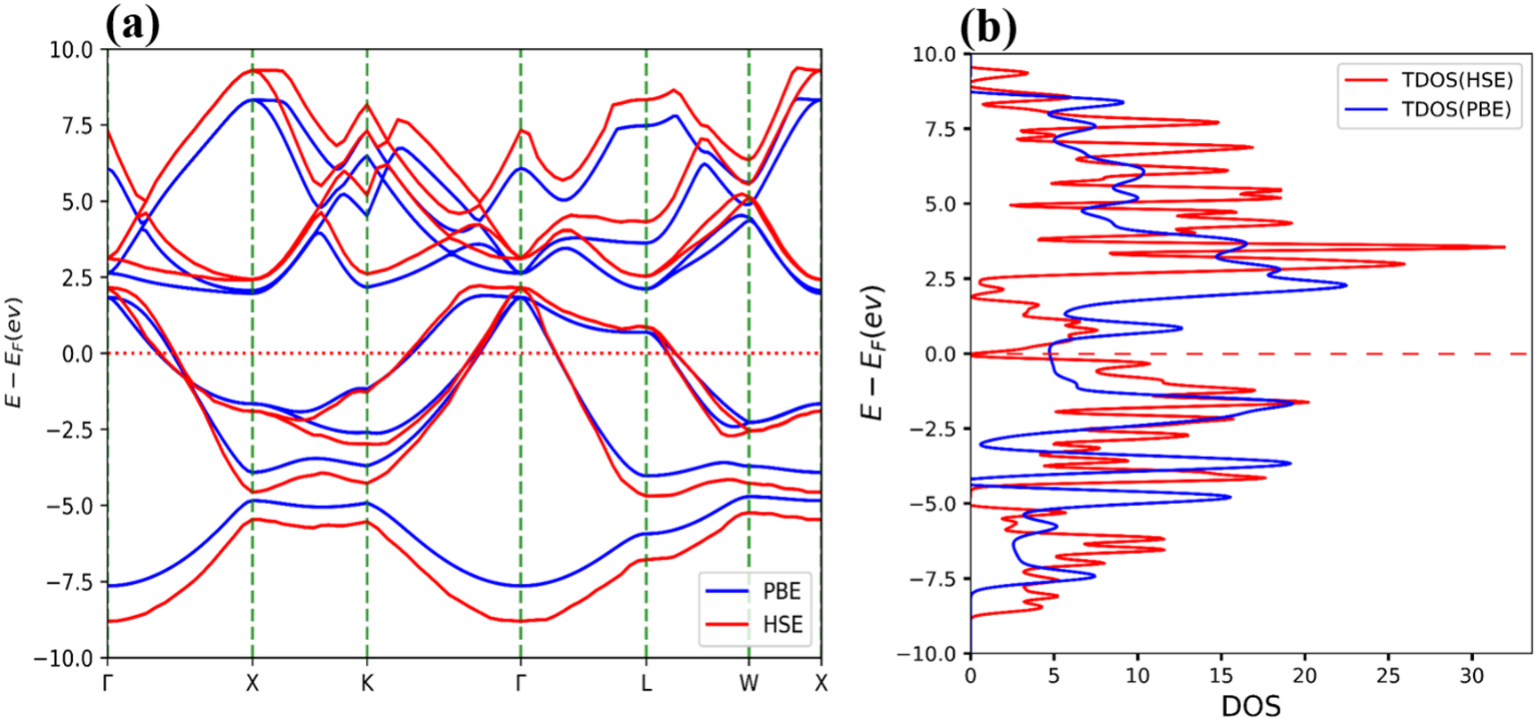
The PBE (blue) and HSE (red) band structure (**a**), and total density of states (TDOS) (**b**) of Li_2_BeAl Heusler alloy. The Fermi level is set to be zero.

**Figure 8:**
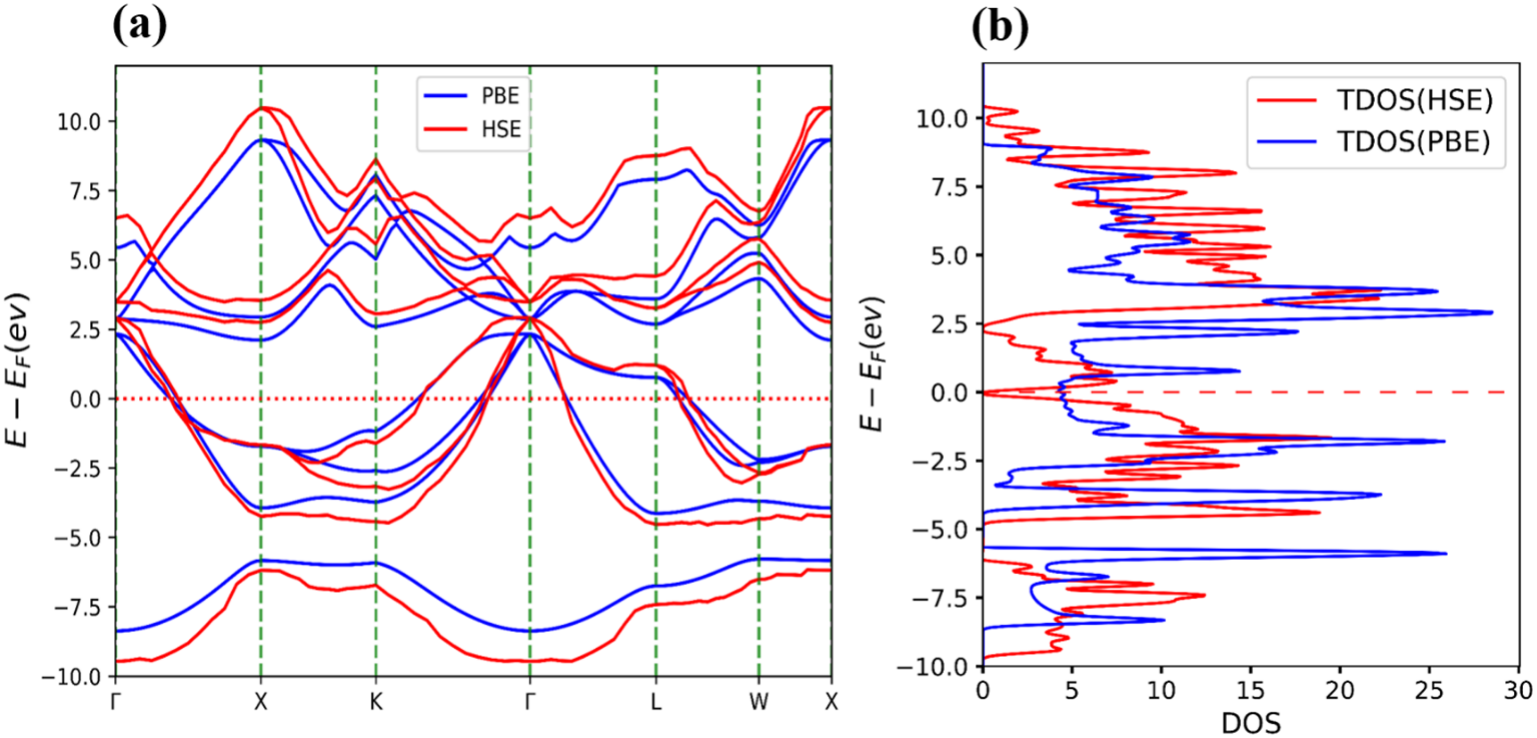
The PBE (blue) and HSE (red) band structure (**a**), and total density of states (TDOS) (**b**) of Li_2_BeGa Heusler alloy. The Fermi level is set to be zero.

This section focuses on the investigation of the electronic properties of Li_2_BeAl and Li_2_BeGa Heusler alloys through the calculation of the electronic band structure and density of states (DOS). Figures 7 and 8 show the band structure and DOSs for both alloys. For the band structure calculation, high-symmetry points (Γ → Χ → Κ → Γ → L → W → X) were considered while the Fermi energy level was set to be 0.0 eV.

As can be seen from these figures, the minimum value of the band gap occurs at X and Γ symmetry points. The values obtained for the indirect band gap using PBE and HSE approximations are 0.13 eV and 0.26 eV, respectively, for Li_2_BeAl composition. The indirect band gap values obtained for the Li_2_BeGa compound were found to be − 0.22 eV and − 0.16 eV from the PBE and HSE approximations, respectively. These results indicate that both alloys have intermetallic properties.

**Figure 9:**
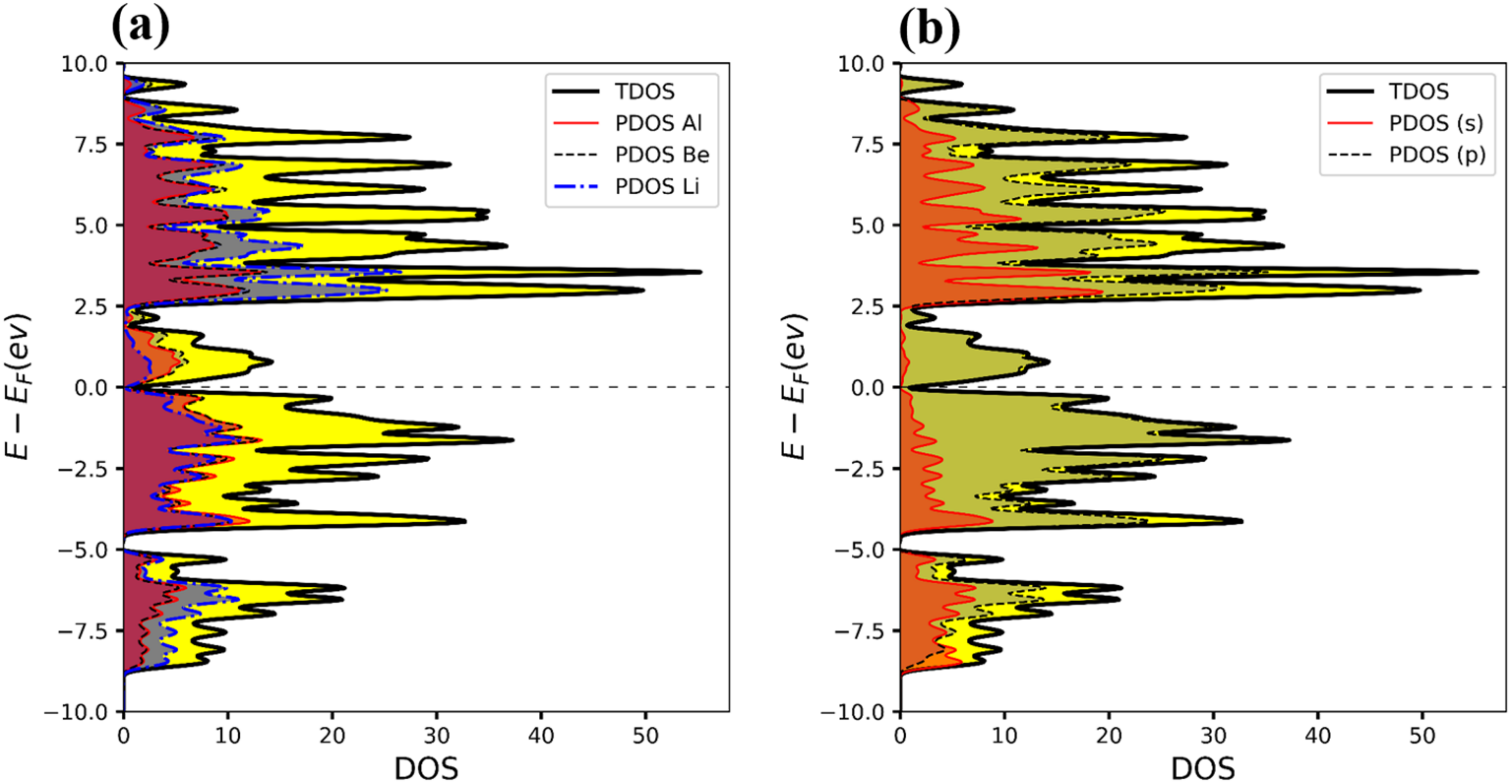
(**a)** Total density of states (TDOS) and projected density of states (PDOS) of atoms, and (**b)** PDOS of orbitals for Li_2_BeAl Heusler alloy. The Fermi level is set to be zero.

**Figure 10:**
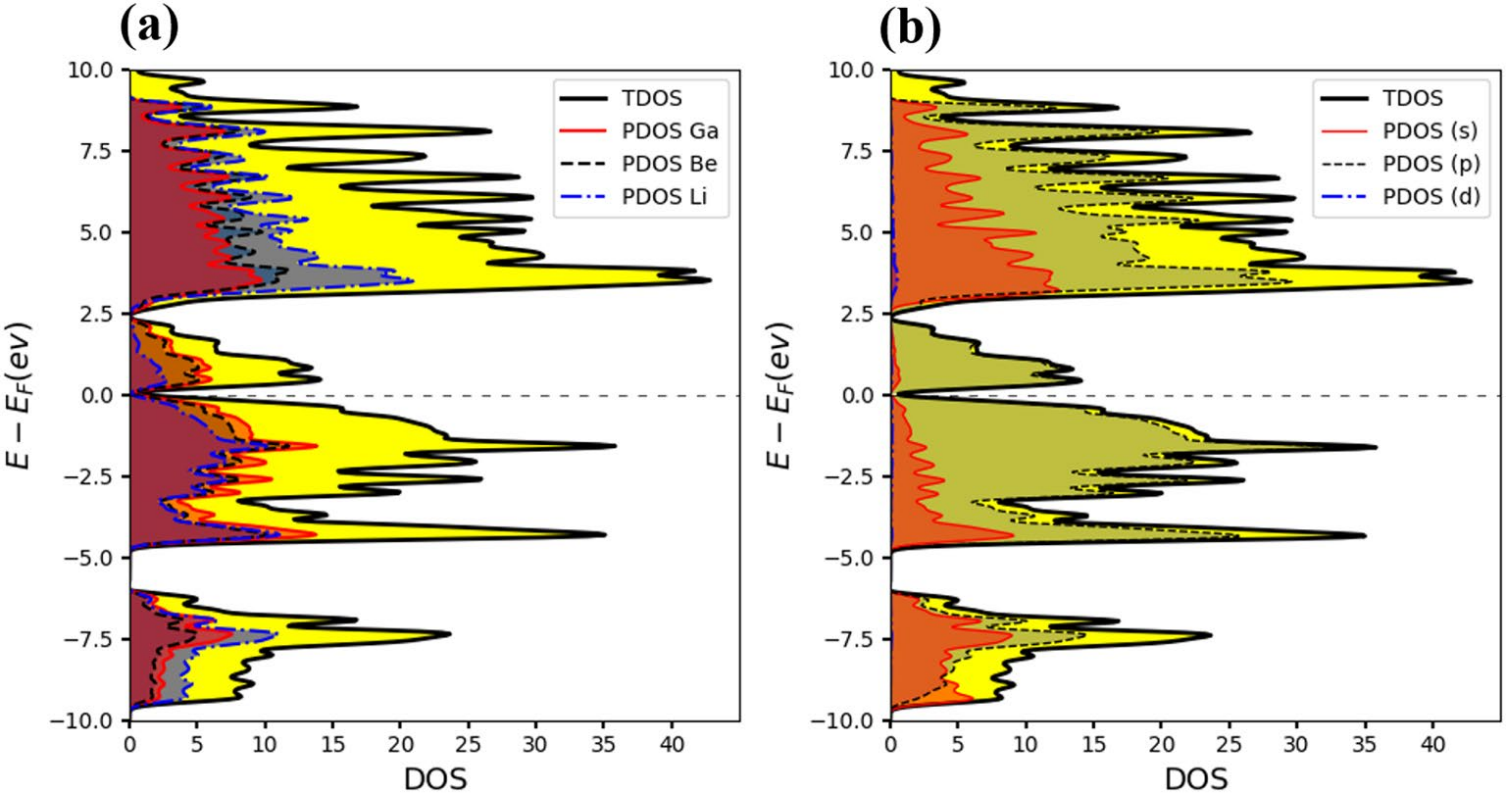
(**a)** Total density of states (TDOS) and projected density of states (PDOS) of atoms, and (**b)** PDOS of orbitals for Li_2_BeGa Heusler alloy. The Fermi level is set to be zero.

To better understand the electronic band structure, the total and projected density of states (PDOS) for both alloys were also plotted. To visualize the contribution of electron orbitals, a Gaussian broadening function of FWHM = 0.05 eV has been applied to broaden the DOS lines. Figure 9a shows that the Li, Be, and Al atoms participate in both valence and conduction bands. Moreover, Fig. 9b shows s-orbital electrons contribute significantly to the conduction band, and p-orbitals have involvement in both conduction and valence bands. Similarly, Fig. 10a shows that the Li, Be, and Ga atoms participate in both valence and conduction bands. Additionally, Fig. 10b shows the s and p-orbitals contribute to the conduction band, while the p-orbital contributes to both valence and conduction bands. These results complement the band structure calculations and show that d-orbital electrons have less contribution to the conduction band.

Maximally-Localized Wannier Functions (MLWF) using the Wannier90 package and the semi-classical Boltzmann transport theory as implemented in Boltztrap2 package have been used to interpolate the band structure and calculate the thermoelectric properties. The interpolated band structure of Wannier90 code with the band structure obtained from the PBE approximation as well as the interpolated band structure of Boltztrap2 with the HSE functional are shown in Figures S.1 and S.2 of the supplementary information. The band structure obtained from the Wannier functions and the semi-classical Boltzmann transport equations are in good agreement with the DFT band structure.

**Figure 11:**
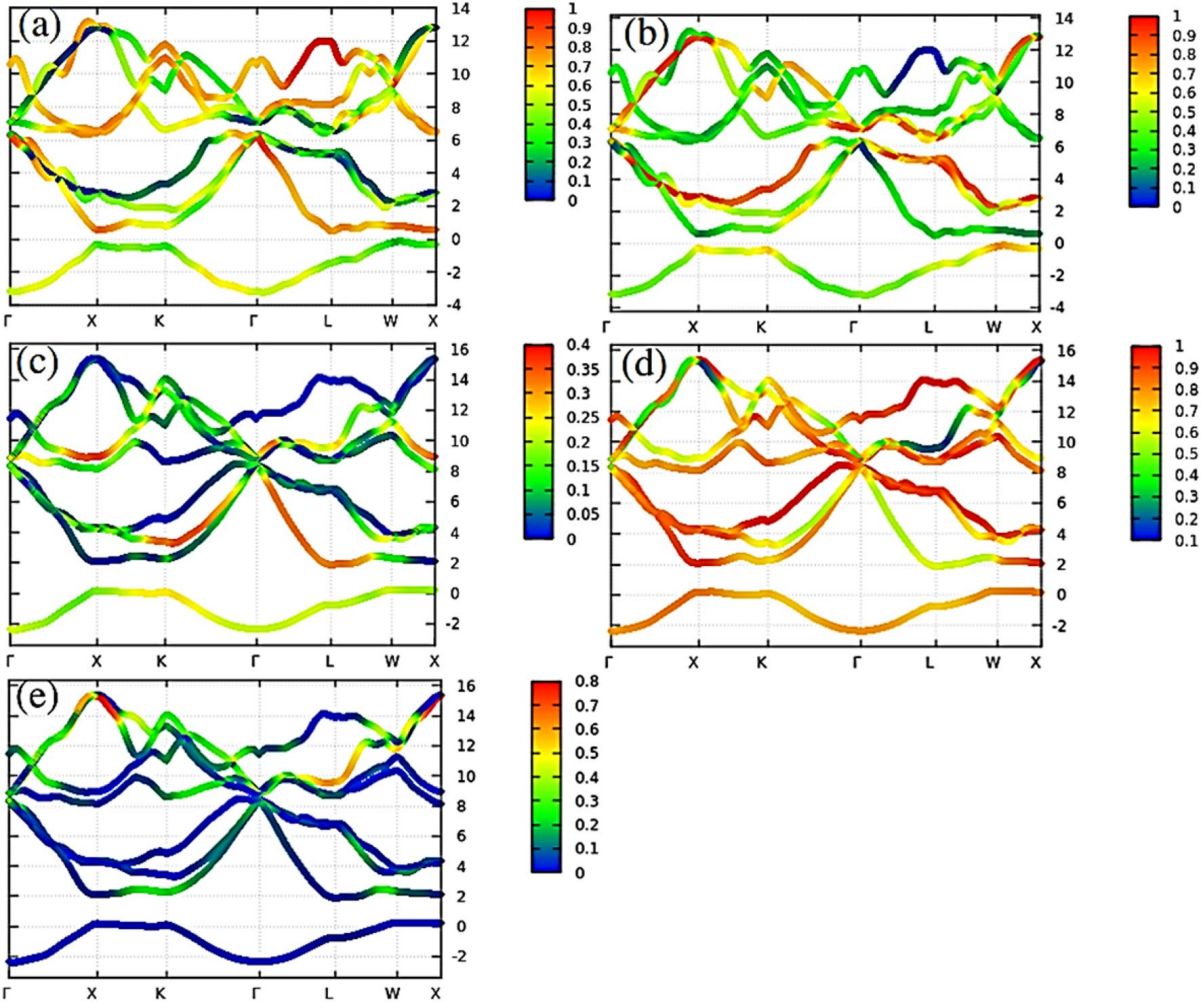
Projected band structure for (**a)** s-orbitals, and (**b)** p-orbitals of Li_2_BeAl Heusler compound, as well as **(c)** s-orbitals and (**d)** p-orbitals, and (**e**) d-orbital for the Li_2_BeGa Full Heusler alloys.

The contributions of s, p, and d orbitals of each band have been calculated through the Wannier90 code for Li_2_BeAl and Li_2_BeGa Heusler alloys. Figure 11 shows that s and p-orbital electrons have significant contributions to the conduction band and p-orbital have greater involvement in both bands. The results from the Wannier functions complement the DOS and band structure calculations.

### Thermoelectric properties

Currently, the world is facing an energy crisis, and there is a need for suitable and efficient alternatives of fossil fuels that are compatible with the environment. Suitable thermoelectric materials can be an adequate substitute for fossil fuels by converting waste energy into electricity. The efficiency of converting thermal energy into electricity in thermoelectric materials depends on the transport coefficients, namely Seebeck coefficient(S), electrical conductivity (σ), thermal conductivity (κ_*e*_ + κ_*l*_), and figure of merit (ZT).

To calculate the thermoelectric properties of Li_2_BeAl and Li_2_BeGa Heusler alloys, the semi-classical Boltzmann transport equation with constant relaxation time and rigid approximation, as implemented in Boltztrap2 code, was used. To test the reliability of the obtained results, the Seebeck coefficient has also been calculated with Maximally-Localized Wannier Functions using the Wannier90 code, and compared with the results of the Boltztrap2 code in Fig. S.3 of the supplementary information. Calculated Seebeck coefficient from two calculation methods are in good agreement, which confirms the validity of the data.

Furthermore, Boltztrap2 package was implemented to calculate thermoelectric properties such as Seebeck coefficient(S), electronic thermal conductivity (κ_e_), electrical conductivity (σ), and the power factor (PF). These properties were calculated as functions of chemical potential, temperature, and carrier concentration using the HSE functional. The results are given in Figs. S.4 to S.9 of the supplementary information.

S, σ, κ_*e*_, and PF for Li_2_BeAl alloy are shown in Fig. S.4 as a function of chemical potential (µ) at constant temperatures of 300, 500, 700, and 900 K. The thermoelectric properties of Li_2_BeGa alloy are also shown in Fig. S.5. The chemical potential (µ) is a crucial parameter affects the transports features of a material. Indeed, the electrons in the valence or conduction band that participate in the electronic transport are determined by the position of µ in the band structure, which affects both the conductivity and the Seebeck coefficient. In Fig. S.4a, two peaks at µ = − 1.32 eV and − 1.12 eV can be seen, and the S quickly tends to be zero when moving away from this range. One of the peaks was related to the p-type (hole with µ < 0) and the other is related to the n-type (electrons with µ > 0) transport conditions. As can be seen, these peaks are very close to the maximum of the valence band and the minimum of the conduction band, respectively. Similar results for Li_2_BeGa alloy can also be seen in Fig. S.5a. In general, with an increase in the temperature the S decreases for both alloys, due to an increase in the thermal energy (i.e., an increase in the electron/hole carrier concentration). The S values of Li_2_BeGa alloy is higher than Li_2_BeAl, so that, the highest value of the S is 929 µV/K at T = 300 K and decreases to 341 µV/K at T = 900 K for Li_2_BeAl. Corresponding values for Li_2_BeGa are 1580 and 859 µV/K, respectively. These values confirms that both compounds and especially Li_2_BeGa alloy have good thermoelectric performance. It is also noticeable mentioning that, the semiconductor nature of the Li_2_BeGa alloy makes the S to be zero for certain values of the chemical potential at low temperatures, which depends on the electrical band gap, and was observable at 300 K.

The Seebeck coefficient (S) in Boltzmann transport calculations is independent of the relaxation time, τ, but the electrical conductivity (σ) is linearly dependent on the τ, under the relaxation-time approximation. Additionally, the Wiedemann–Franz equation, κ_e_ = σLT (L: Lorentz factor, 2.45 × 10^–8^ WΩ/Κ^2^), yields the electrical thermal conductivity (κ_*e*_); hence κ_*e*_ and τ are related. Because of the intricate dispersion mechanism, the relaxation times in bulk materials are typically difficult to ascertain. Yabuuchi et al.^48^, however, used a fixed value of τ = 1 × 10^–14^ s after comparing the computed results with the experimental values, which is also adopted in this work.

In Figs. S.4b and S.5b, unlike S, the σ exhibit an almost similar behavior at different temperatures. Also, the σ was zero in the range of µ = − 1.7 to − 0.8 eV at all temperatures for Li_2_BeAl alloy, whereas, for Li_2_BeGa compound the zero σ values are observed in the µ = − 0.93 to 2.15 eV range. Also, the behavior of σ against µ was completely opposite to S, so that, where S is maximum σ has the lowest value, and vice versa. Also, by moving away from the Fermi level, the σ increases, because, the σ is directly proportional to the charge carrier density. Also, in both alloys, σ for n-type doping interval (positive μ values) is higher than the p-type doping (negative μ values).

Figures S.4c and S.5c show κ_e_ as a function of µ at constant temperatures of 300, 500, 700, and 900 K. κ_e_ specifies the ability to transfer heat by electrons and phonons. The electronic thermal conductivity depends on the electrical conductivity based on the Wiedemann–Franz Law. As can be seen in these figures, unlike σ which behaved similarly at all temperatures, the electronic thermal conductivity increases significantly with increasing the temperature from 300 to 900 K. The lowest value of κ_e_ for Li_2_BeAl alloy is in the range of µ = − 1.7 to − 0.8 eV for all temperatures, whereas, for Li_2_BeGa alloy lowest value of κ_e_ are in the range of µ = − 0.95 to 2.2 eV. In both alloys the κ_e_ of positive µ is higher than negative µs, which indicate that n-type doping results in a higher κ_e_ than p-type doping. Because, the better the thermoelectric performance of the material was obtained at lower κ_e_, p-doping is more favorable in both alloys.

Figures S.4d and S.5d shows the power factor (PF) as a function of chemical potential. PF is one of the important quantities to check the efficiency of a thermoelectric material, and can be obtained from the calculated values of S and σ (PF = S^2^σ). As can be seen, PF increases with increasing the temperature, and the lowest value of PF was observed at T = 300 K. For Li_2_BeAl alloy, two peaks of 0.015 and 0.2 W/mk^2^ at T = 900 K can be seen in the p-type doping region, namely, µ = − 2.05 and − 0.22 eV. However, for Li_2_BeGa alloy, the maximum values of PF at T = 900 K are 0.016 and 0.013 W/mK^2^ which were observed at µ = 2.81 and -1.07 eV, respectively. Also, in contrast to the Al containing alloy, Li_2_BeGa alloy shows higher PF values for n-type doping, so, to have a better thermoelectric performance, electron doping will be better than hole doping in this alloy.

The thermoelectric properties of Li_2_BeAl and Li_2_BeGa alloys were shown as a function of carrier concentration in Figs. S.6 and S.7, respectively. The positive values of the carrier concentration correspond to the hole doping (p-type) and the negative values correspond to the electron doping (n-type) in these figures. Figures S.6a and S.7a show S as a function of carrier concentration. S has a strong dependence on the carrier concentration of the charge. As can be seen, two peaks were obtained for S, one peak has a positive value and the other one has a negative value. Both peaks for both alloys are observed in electron doping region (i.e., carrier concentration of ~ 2 × 10^22^ cm^-3^), however, the magnitude of S for Li_2_BeAl alloy is greater than that of the Li_2_BeGa.

Figures S.6b and S.7b show the electrical conductivity as a function of carrier concentration. It is seen that, in both Heusler alloys n-type doping has more σ than p-type doping. The carrier concentration dependence of the electrical thermal conductivity was shown in Figures S.6c and S.7c. As can be seen, the obtained values of κ_e_ in n-type doping is more than p-type doping for both alloys. Furthermore, both σ and κ_e_ values in the n-type doping region are approximately zero at the carrier concentrations of − 1.92 × 10^22^ and − 2.0 × 10^22^ cm^-3^ for Li_2_BeAl and Li_2_BeGa alloys, respectively. PF was also plotted versus carrier concentration in Figures S.6d and S.7d. The maximum values of PF were obtained in the n-type doping region and T = 900 K for both alloys.

The temperature dependence of the thermoelectric properties for Li_2_BeAl and Li_2_BeGa compounds were shown in Figures S.8 and S.9, respectively, at fixed carrier concentrations for hole and electron doping. Positive and negative S values indicate p-type and n-type doping, respectively. It is observed that S increases rapidly with increasing the temperature. The maximum value of |S| was obtained at the temperatures of about 850 and 1000 K for Li_2_BeAl and Li_2_BeGa compounds, respectively. The obtained results are in good agreement with the reported results for LiScGe composition^49^.

κ_e_ and σ also increased with increasing temperature in both alloys, because electrons receive more energy and their flux increases with the temperature raise. Also, the non-linear behavior of σ with temperature indicates that both alloys are semi-metallic. Power factor (PF) results can also be seen in Figs. S.8d and S.9d. It has a behavior similar to that of the Seebeck coefficient and increases with increasing the temperature. For electron doping, it reaches a maximum value of 0.030 and 0.014 mW/mK^2^ at 1000 K for Li_2_BeAl and Li_2_BeGa alloys. For hole doping, PF increases with increasing the temperature to reach maximum values of 0.004 and 0.003 mW/mK^2^ for Li_2_BeAl and Li_2_BeGa alloys at 1000 K, indicating that for both Li_2_BeAl and Li_2_BeGa alloys, electron doping performance was more profound than hole doping. Furthermore, PF shows the performance of a thermoelectric material and the higher PF values obtained for Li_2_BeAl alloy show the better performance of this compound as a thermoelectric material.

**Figure 12:**
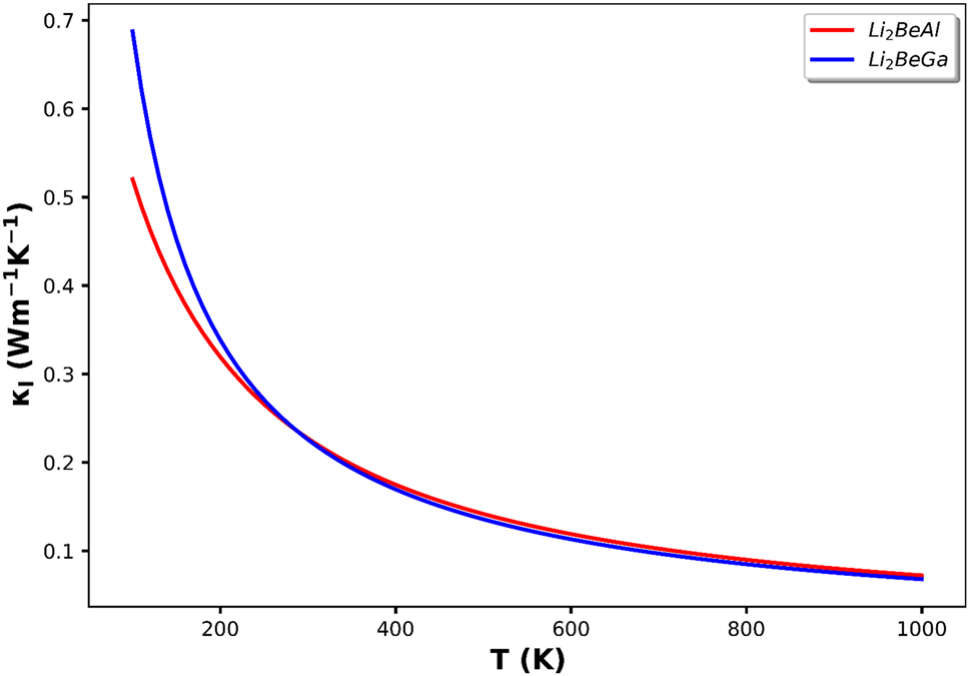
Lattice thermal conductivity as a function of temperature for Li_2_BeAl (blue) and Li_2_BeGa (red) Full Heusler alloys.

Lattice thermal conductivity (κ_*l*_) was also calculated as a function of temperature for Li_2_BeAl and Li_2_BeGa alloys. The results shown in Fig. 12 exhibit that κ_*l*_ decreases with increasing the temperature due to the phenomenon of phonon scattering and reaches its lowest value at temperatures above 800 K, which is in good agreement with the results obtained for Li_2_BeSi, Li_2_BeGe, Li_2_BeSn alloys^50^. Also, Li_2_BeGa alloy has higher thermal conductivity than Li_2_BeAl.

The performance of a thermoelectric material and its capacity to effectively produce electrical energy from a thermal source are evaluated by the figure of merit (ZT). For appropriate thermoelectric applications, materials with a ZT close to or greater than unity are sufficient and viable options. ZT can be calculated using,

3 $$ZT = {{S^{2} T\sigma } \mathord{\left/ {\vphantom {{S^{2} T\sigma } {\left( {\kappa_{e} + \kappa_{l} } \right)}}} \right. \kern-0pt} {\left( {\kappa_{e} + \kappa_{l} } \right)}}$$

**Figure 13:**
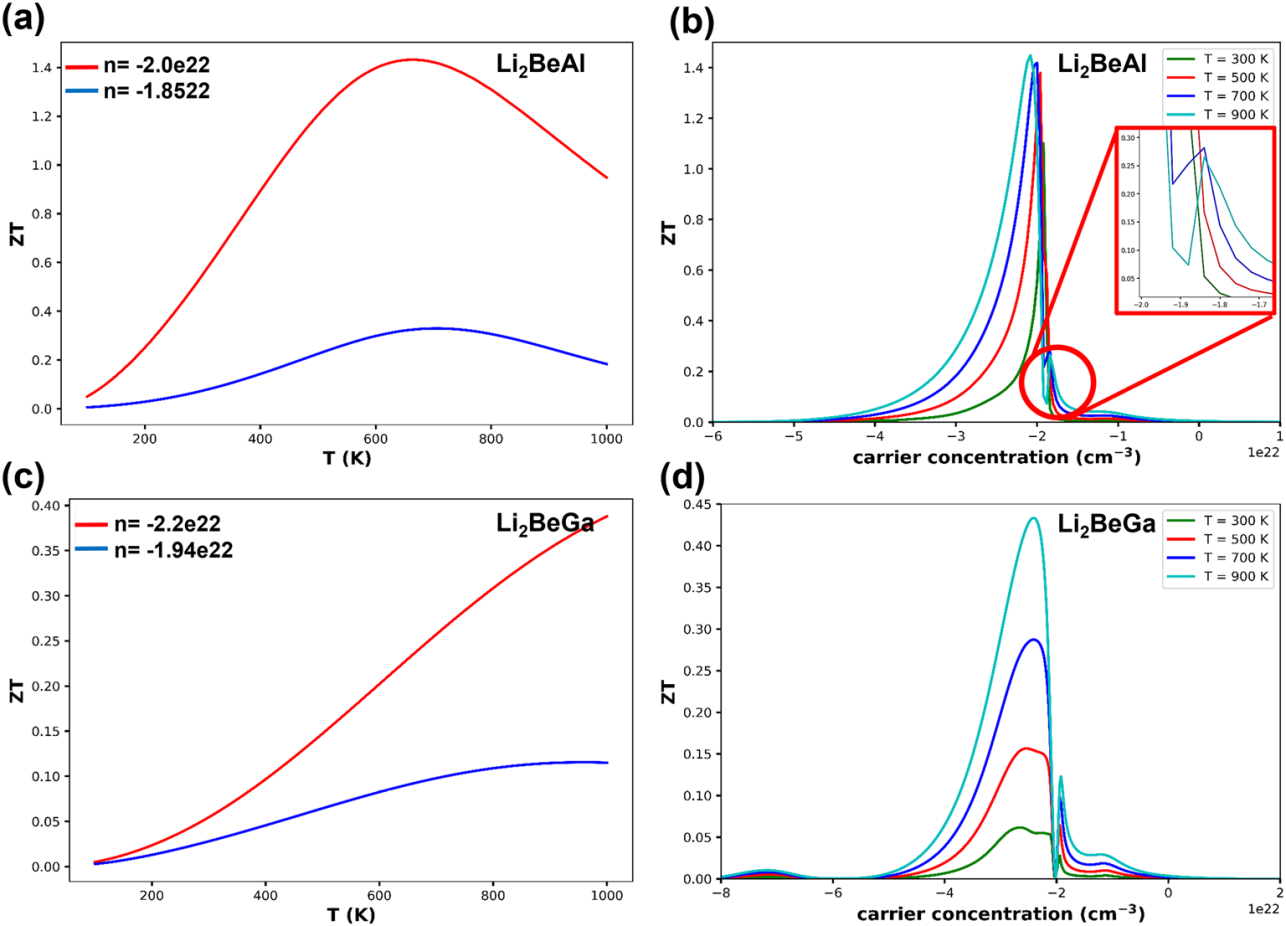
The figure of merit as a function of temperature (left), and carrier concentration (right) for Li_2_BeAl (**a** and **b**) Li_2_BeGa (**c** and **d**) Full Heusler alloys.

Figure 13 represent the calculated ZT for Li_2_BeAl and Li_2_BeGa Heusler alloys as a function of temperature at constant carrier concentrations. The maximum value of ZT for n-type doping is 1.43 at 660 K for Li_2_BeAl alloy, and is 0.39 at 1000 K for Li_2_BeGa alloy. However, for p-type doping the maximum ZT values of 0.33 for Li_2_BeAl alloy at 710 K and 0.10 for Li_2_BeGa alloy at 950 K were obtained. The obtained results show that electron doping in both alloys have better efficiency than hole doping and Li_2_BeAl alloy is better than Li_2_BeGa alloy for use in thermoelectric devices.

Figure 13b and d also show ZT as a function of carrier concentration for Al and Ga containing alloys at different temperatures. Kindly check and confirm inserted volume number is correct for the reference^2^. In this figure, it is seen that the ZT obtained for electron doping has higher values than hole doping in both alloys.

## Conclusion

The crystal structure, electronic, elastic, and thermoelectric properties of Li_2_BeAl and Li_2_BeGa Heusler alloys were investigated using DFT and Boltzmann transition theory methods. Results show that compounds in the inverse Heusler structure are more energetically stable than their full Heusler structure counterparts, and these compounds are also mechanically and chemically stable. The electronic properties of both alloys reveal an intermetallic behavior. Car-Parrinello ab-initio molecular dynamics simulations confirm the thermal stability of both crystals at T = 1000 K, and therefore, the thermoelectric properties were calculated for both alloys up to 1000 K. Electronic thermal conductivity increased with temperature, and n-type doped alloys exhibited higher electronic thermal conductivity. The higher values of power factor (PF) and figure of merit (ZT) for electron doping, confirm that electron doping is more profound than hole-doping in both compounds. At T = 300 K for electron doping the ZT of 0.68 and 0.05 were obtained for Li_2_BeAl and Li_2_BeGa alloys, at carrier concentration of − 2.2 × 10^22^ cm^-3^. However, the considerable value of ZT = 1.43 for n-doped Li_2_BeAl at T = 660 K shows the great potential of this compound in design of the thermoelectric materials. The potential use of both Li_2_BeAl and Li_2_BeGa Heusler alloys in spintronic devices and thermoelectric materials is suggested based on the findings.

### Supplementary Information

Supplementary Figures.

## Data Availability

All data generated or analyzed during this study are included in this published article.
