# Supplementary material for: Crystal structure, stability, and transport properties of Li2BeAl and Li2BeGa Heusler alloys: a DFT study
Source: Sci Rep. 2024 May 28;14:12201. doi: 10.1038/s41598-024-63092-x (PMC11133474; doi:10.1038/s41598-024-63092-x)
Supplement: Supplementary file 1 — Supplementary Figures. [file 41598_2024_63092_MOESM1_ESM.pdf]

Supplementary Information

For

Crystal Structure, Stability, and Transport Properties of Li_2BeAl and Li_2BeGa Heusler Alloys: A DFT Study

Sima Mahmoudi,[a,b] Mir Maqsood Golzan,[a] Ebrahim Nemati-Kande*[b]

[a] *Department of Physics, Faculty of Sciences, Urmia University, Urmia, Iran*
[b] *Department of Physical Chemistry, Chemistry Faculty, Urmia University, Urmia, Iran*

*Corresponding author.
E-mail address: e.nemati@urmia.ac.ir

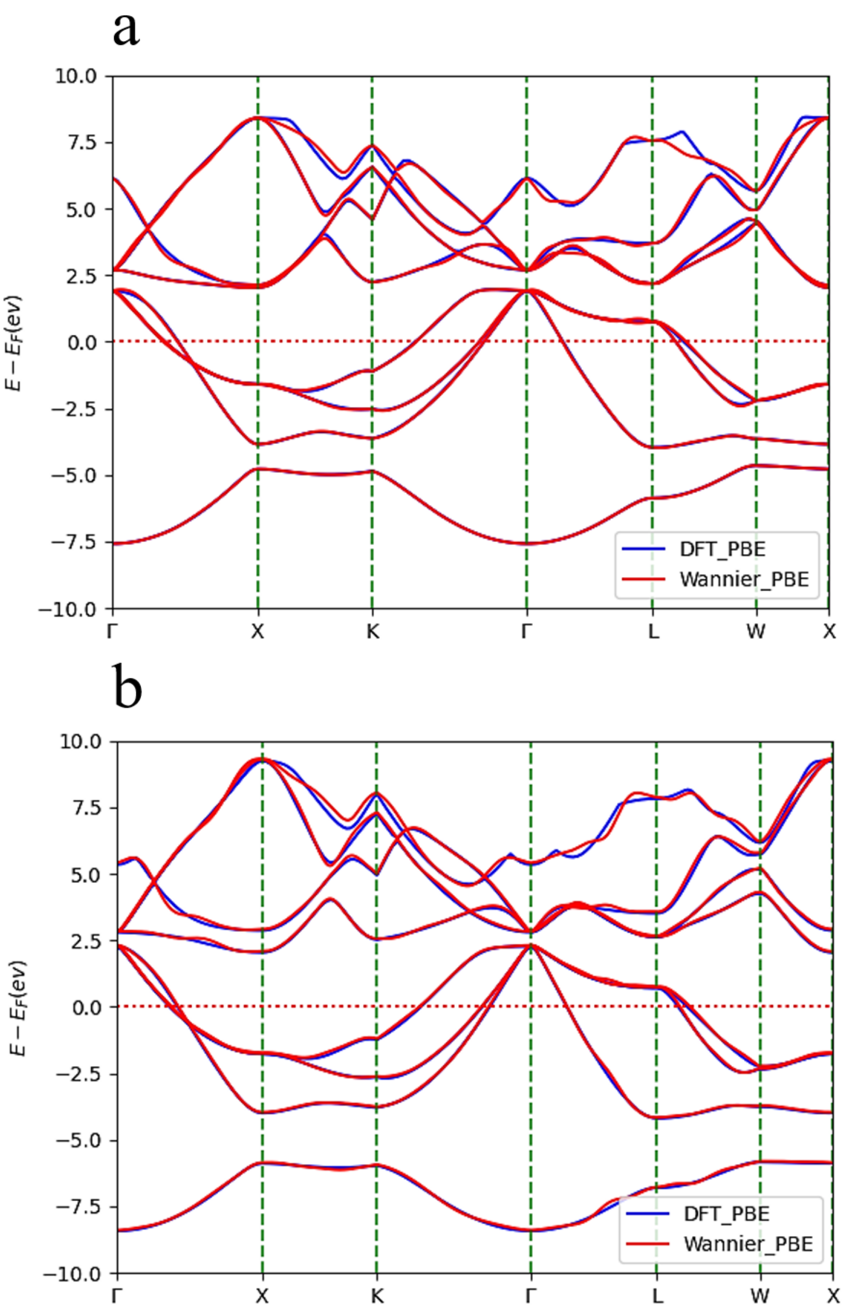

Fig. S.1. Electronic band structure of (**a**) Li_2BeAl and (**b**) Li_2BeGa Heusler alloys. DFT-PBE bands (blue lines) are compared with Wannier-PBE (red lines). The Fermi level is set to be zero.

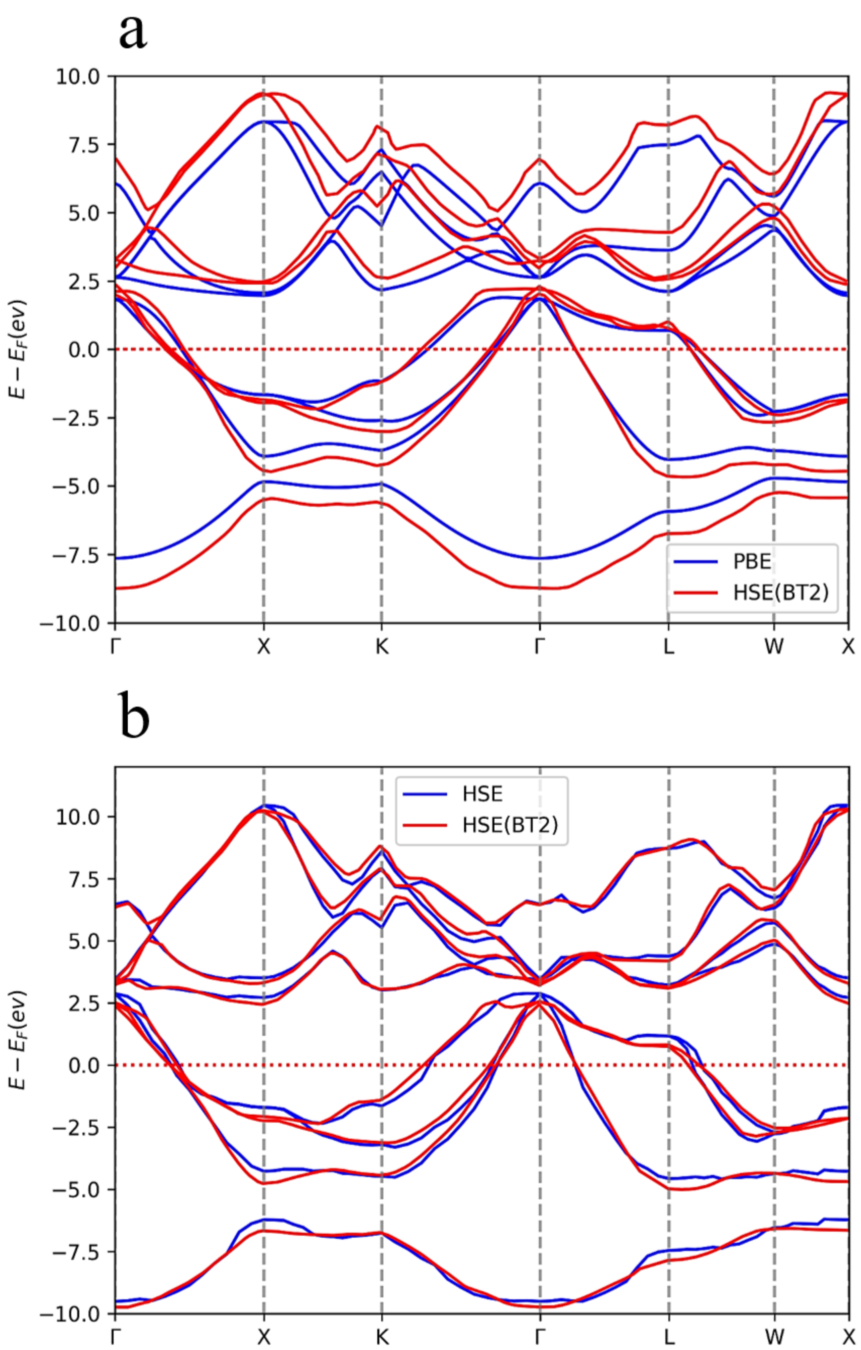

Fig. S.2. Electronic band structure of (**a**) Li_2BeAl and (**b**) Li_2BeGa Heusler alloys. DFT-HSE bands (blue lines) are compared with Boltztrap2-HSE (red lines). The Fermi level is set to be zero.

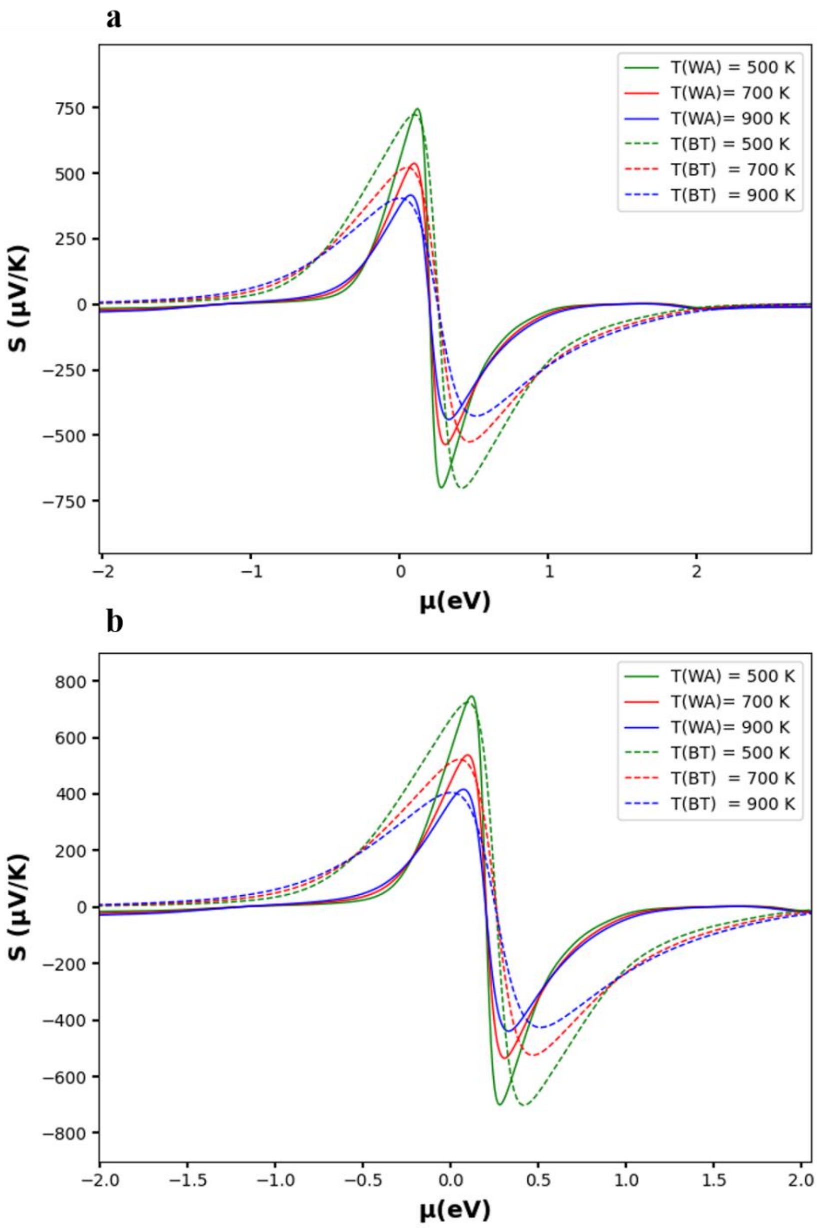

Fig. S.3. The Seebeck coefficients for (**a**) Li_2BeAl and (**b**) Li_2BeGa Heusler alloys obtained from Wannier90 (WA) and Botztrap2 (BT) softwares at different temperatures using PBE functional.

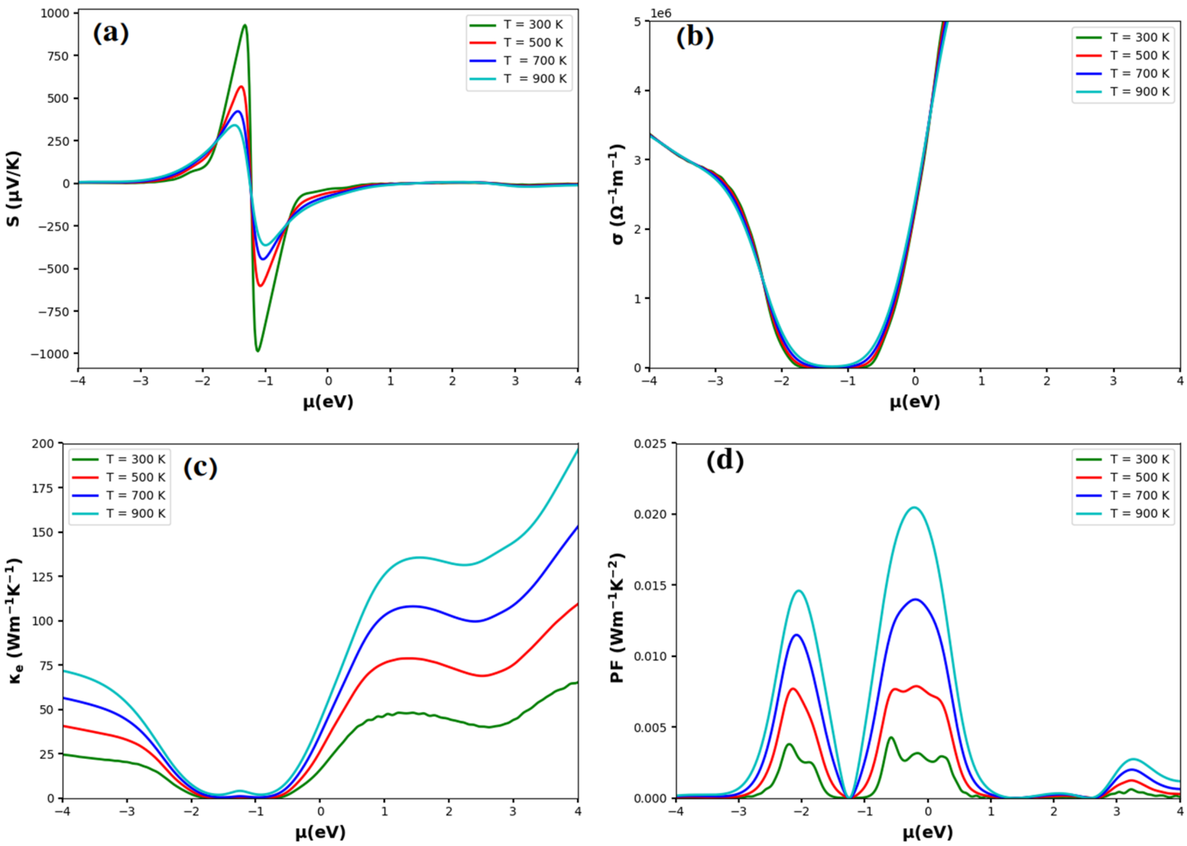

Fig. S.4. Thermoelectric properties as a function of chemical potential for Li_2BeAl Full Heusler alloy obtained from PBE functional. (**a**) Seebeck coefficient, (**b**) Electrical conductivity, (**c**) Electronic thermal conductivity, (**d**) power factor.

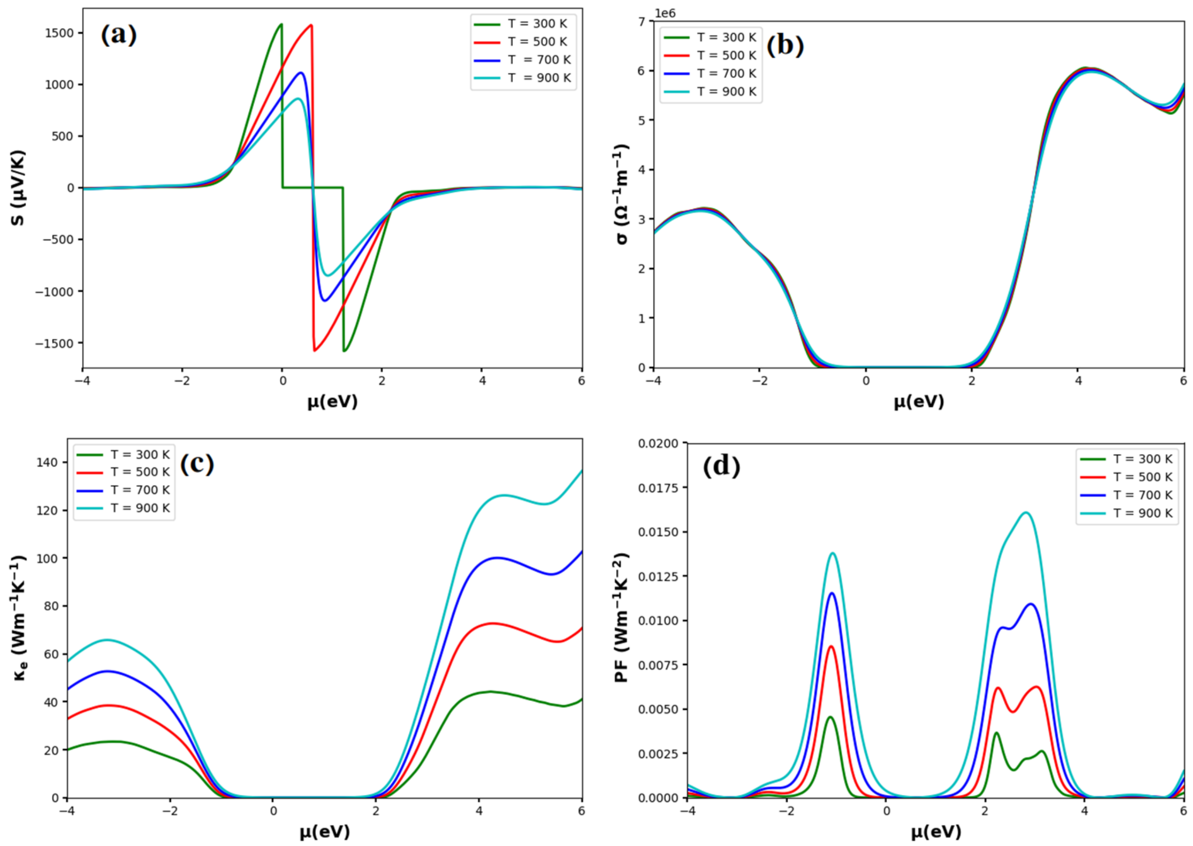

Fig. S.5. Thermoelectric properties as a function of chemical potential for Li_2BeGa Full Heusler alloy obtained from PBE functional. (**a**) Seebeck coefficient, (**b**) Electrical conductivity, (**c**) Electronic thermal conductivity, (**d**) power factor.

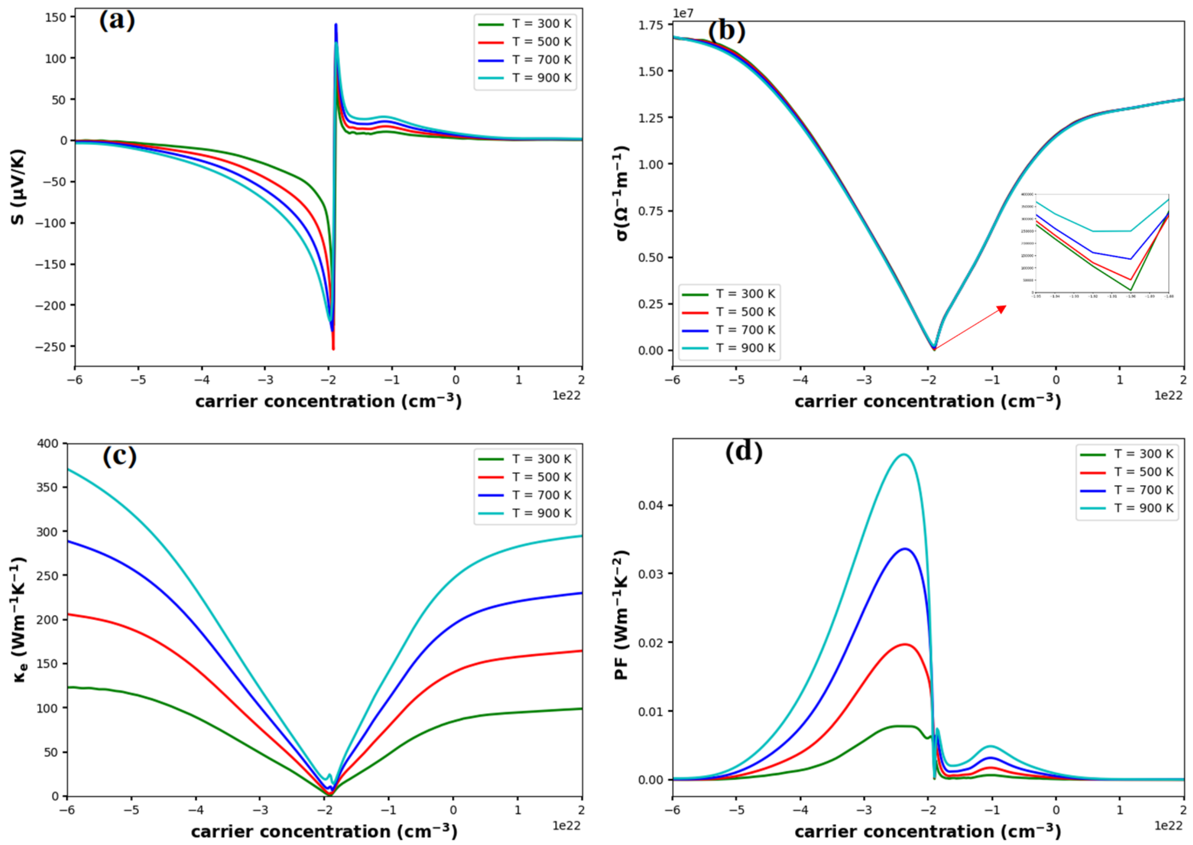

Fig. S.6. Thermoelectric properties as a function of carrier concentration Li_2BeAl Full Heusler alloy obtained from PBE functional. (**a**) Seebeck coefficient, (**b**) Electrical conductivity, (**c**) Electronic thermal conductivity, (**d**) power factor.

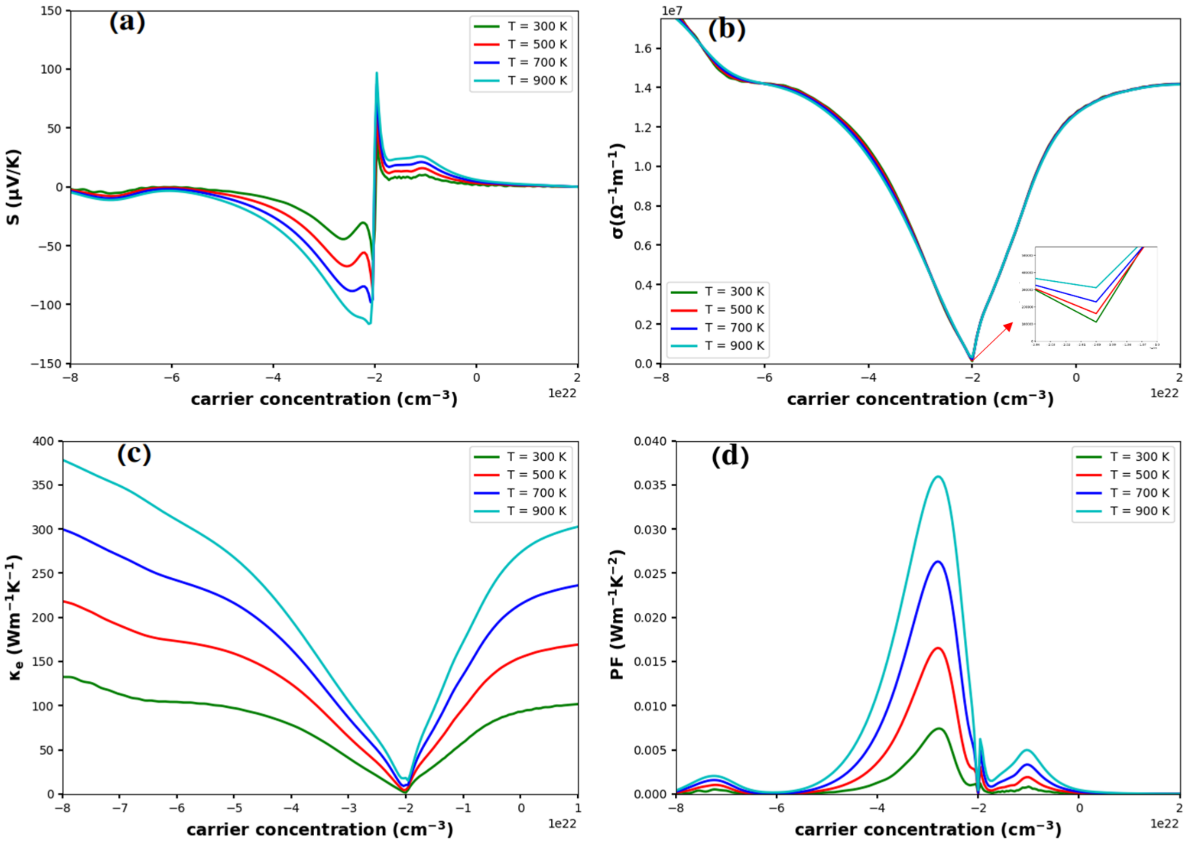

Fig. S.7. Thermoelectric properties as function of carrier concentration for Li_2BeGa Full Heusler alloy obtained from PBE functional. (**a**) Seebeck coefficient, (**b**) Electrical conductivity, (**c**) Electronic thermal conductivity, (**d**) power factor.

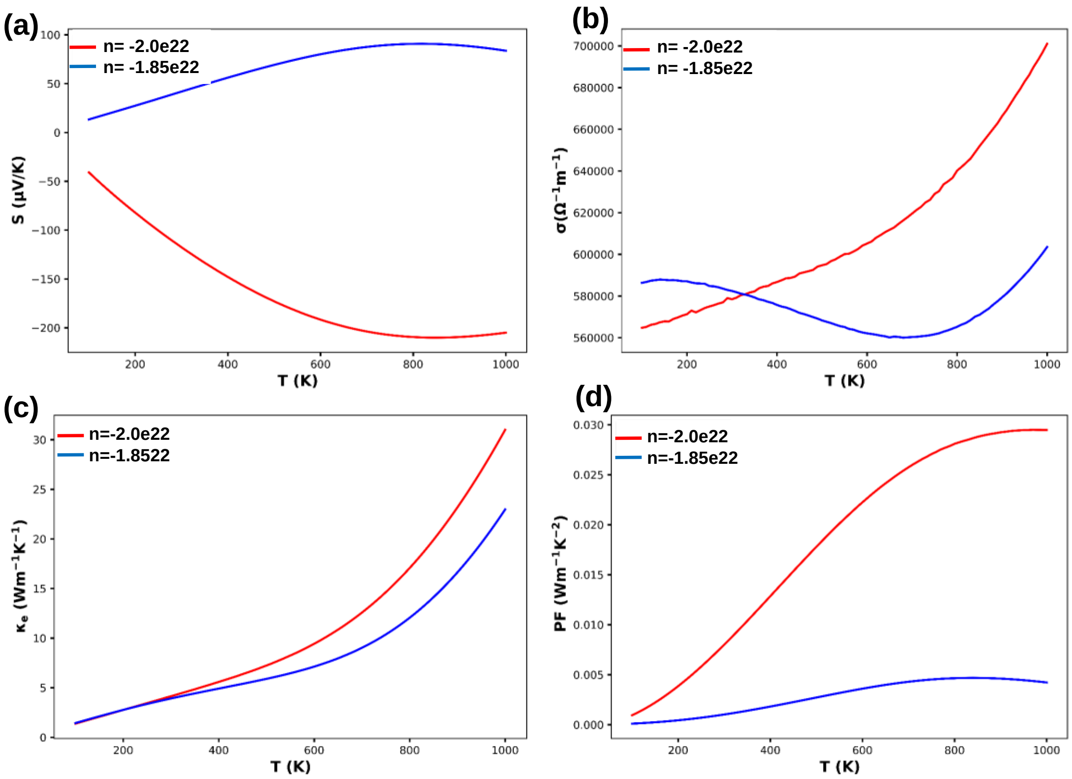

Figure S.8. (**a**) Seebeck coefficient, **(b)** Electrical conductivity, (**c**) Electronic thermal conductivity, and (**d**) power factor as a function of temperature for Li_2BeAl Full Heusler alloys

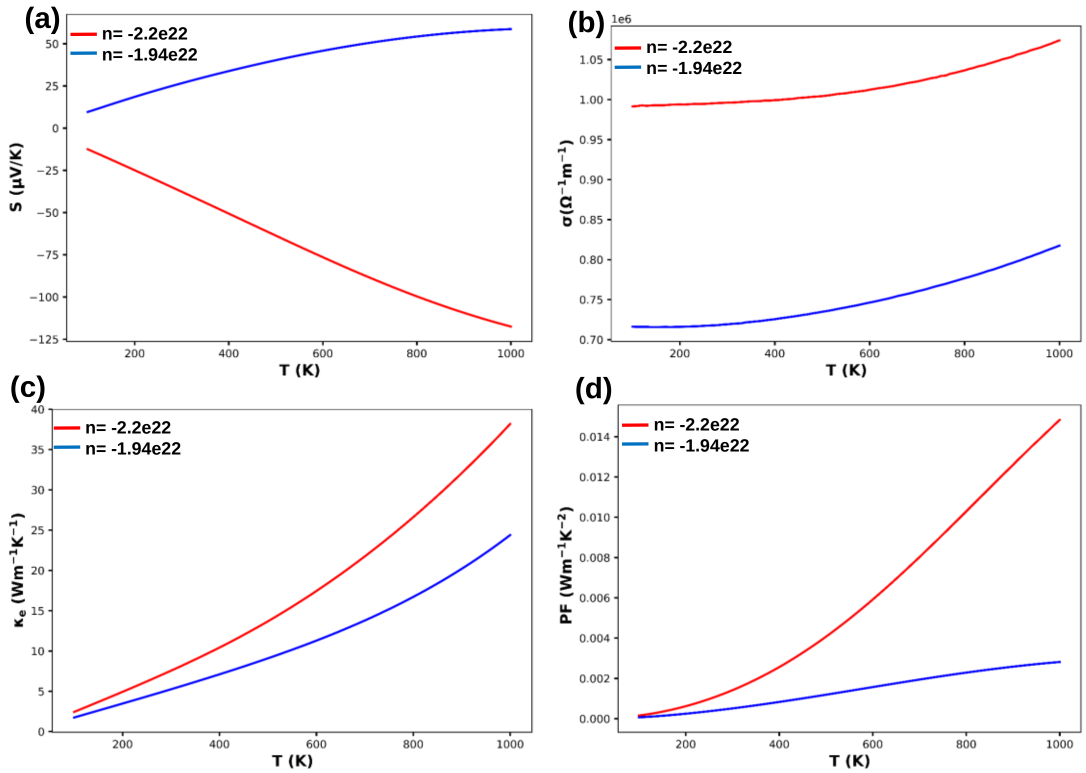

Figure S.9. (**a**) Seebeck coefficient, (**b**) Electrical conductivity, (**c**) Electronic thermal conductivity, and (**d**) power factor as a function of temperature for Li_2BeGa Full Heusler alloys.
